# Trait sensitivity to positive feedback is a predisposing factor for several aspects of compulsive alcohol drinking in male rats: behavioural, physiological, and molecular correlates

**DOI:** 10.1007/s00213-023-06460-1

**Published:** 2023-09-08

**Authors:** Agata Cieslik-Starkiewicz, Karolina Noworyta, Joanna Solich, Agata Korlatowicz, Agata Faron-Górecka, Rafal Rygula

**Affiliations:** 1 Department of Pharmacology, Affective Cognitive Neuroscience Laboratory, Maj Institute of Pharmacology Polish Academy of Sciences, 12 Smetna Street, 31-343 Krakow, Poland; 2 Department of Pharmacology, Biochemical Pharmacology Laboratory, Maj Institute of Pharmacology Polish Academy of Sciences, 12 Smetna Street, 31-343 Krakow, Poland

**Keywords:** Feedback Sensitivity, Animal model, Alcohol, Rat

## Abstract

**Introduction:**

Alcohol use disorder (AUD) is one of the most common psychiatric disorders and a leading cause of mortality worldwide. While the pathophysiology underlying AUD is relatively well known, the cognitive mechanisms of an individual’s susceptibility to the development of alcohol dependence remain poorly understood. In this study, we investigated the theoretical claim that sensitivity to positive feedback (PF), as a stable and enduring behavioural trait, can predict individual susceptibility to the acquisition and maintenance of alcohol-seeking behaviour in rats.

**Methods:**

Trait sensitivity to PF was assessed using a series of probabilistic reversal learning tests. The escalation of alcohol intake in rats was achieved by applying a mix of intermittent free access and instrumental paradigms of alcohol drinking. The next steps included testing the influence of sensitivity to PF on the acquisition of compulsive alcohol-seeking behaviour in the seeking-taking punishment task, measuring motivation to seek alcohol, and comparing the speed of extinction and reinstatement of alcohol-seeking after a period of abstinence between rats expressing trait insensitivity and sensitivity to PF. Finally, trait differences in the level of stress hormones and in the expression of genes and proteins in several brain regions of interest were measured to identify potential physiological and neuromolecular mechanisms of the observed interactions.

**Results:**

We showed that trait sensitivity to PF in rats determines the level of motivation to seek alcohol following the experience of its negative consequences. They also revealed significant differences between animals classified as insensitive and sensitive to PF in their propensity to reinstate alcohol-seeking behaviours after a period of forced abstinence. The abovementioned effects were accompanied by differences in blood levels of stress hormones and differences in the cortical and subcortical expression of genes and proteins related to dopaminergic, serotonergic, and GABAergic neurotransmission.

**Conclusion:**

Trait sensitivity to PF can determine the trajectory of alcohol addiction in rats. This effect is, at least partially, mediated via distributed physiological and molecular changes within cortical and subcortical regions of the brain.

**Supplementary Information:**

The online version contains supplementary material available at 10.1007/s00213-023-06460-1.

## Introduction

Alcohol use disorder (AUD) is a chronic disease that is characterized by gradual escalation of alcohol consumption over time and a compulsive alcohol-seeking behaviour persisting despite negative consequences. While the pathophysiology underlying AUD is relatively well known, the cognitive mechanisms of an individual’s susceptibility to the development of alcohol dependence remain poorly understood. One of the proposed cognitive phenotypes that is intermediate to AUD is aberrant sensitivity to reinforcement. Indeed, several studies have demonstrated a relationship between positive reinforcement sensitivity, particularly in relation to fun-seeking, and higher alcohol intake (Feil and Hasking 2008; Franken and Muris 2006; Loxton and Dawe 2001). On the other hand, decreased sensitivity to positive reinforcement was associated with the presence of negative affectivity, resulting in alcohol self-medicating as a way to alleviate negative emotional states (Heinz et al. 2009; Stewart et al. 2011; Veilleux et al. 2014). While many different approaches have been used to probe sensitivity to positive reinforcement, very few of them have tested it in the complex cognitive context, and even fewer have allowed for translational comparisons between humans and animal models.

One of the most effective, ecologically valid, and fully translational methods of measuring an individual’s sensitivity to positive reinforcement is the assessment of “win-stay” behaviour in a probabilistic reversal learning (PRL) task (Cools et al. 2002; Paulus et al. 2002, 2003). The PRL involves adapting behaviour to changing stimulus-reward and stimulus-punishment contingencies to maximize reward and minimize punishment under conditions of uncertainty (Rygula et al. 2018). This behavioural paradigm has been successfully applied in research focused on neurochemical and neuroanatomical correlates of reinforcement sensitivity in healthy subjects as well as detecting cognitive deficits in a wide array of pathological states and animal models (Rygula et al. 2018). Recent studies from our laboratory demonstrated that in rodents, sensitivity to positive feedback (PF) is a stable and enduring behavioural trait (Noworyta-Sokolowska et al. 2019) that can affect the sensitivity of rats to the effects of pharmacological treatment (Noworyta and Rygula 2021). Another study revealed that sensitivity to negative feedback can determine the propensity of rats to compulsively drink alcohol (Cieslik et al. 2022).

In the current study, we combined this advanced behavioural technique allowing the determination of sensitivity to PF in rats as a stable and enduring trait, with the examination of the impact of this trait on individual susceptibility to the acquisition and maintenance of alcohol-seeking behaviour. The escalation of ethanol intake in rats was achieved by applying a mix of intermittent free access and instrumental paradigms of alcohol drinking, such as the intermittent access two-bottle choice (2BC) (Cieslik et al. 2022) and seeking-taking (ST) tasks (Giuliano et al. 2018). The next steps included testing the influence of sensitivity to PF on the acquisition of compulsive alcohol-seeking behaviour in the seeking-taking punishment (STP) task and measuring motivation to seek alcohol in the progressive ratio schedule of reinforcement (PRSR) task. Finally, we measured how trait sensitivity to PF affected the extinction and reinstatement of alcohol-seeking after a period of abstinence. To identify potential physiological and neuromolecular mechanisms of the observed interactions between trait sensitivity to PF and the acquisition of compulsive alcohol drinking, we measured trait differences in the levels of stress hormones and the expression of genes and proteins in several brain regions of interest.

## Materials and methods

### Ethical statement

All experiments were conducted in accordance with the European Union guidelines for the care and use of laboratory animals (2010/63/EU). Experimental protocols were reviewed and approved by the 2nd Local Institutional Animal Care and Use Committee, Institute of Pharmacology Polish Academy of Sciences in Krakow. The authors attest that all efforts were made to minimize the number of animals used and their suffering.

### Subjects and housing

We used 40 male Sprague‒Dawley rats (Charles River, Germany) weighing 176–200 g upon arrival. Rats were group-housed (four animals per cage) in an enriched environment (plastic pipes 25 cm long and wooden blocks) under controlled temperature (21 ± 1 °C) and humidity (40–50%) under a 12-h light/dark cycle (lights on at 7:00 AM). Throughout the experiment, rats were mildly food restricted to 85% of their free-feeding weight (according to the normal growth curve recommended by the laboratory rodent supplier—Charles River Research Models and Services Catalogue) by providing 15 g of food pellet/rat/day (standard laboratory chow). Water was always available ad libitum. All behavioural procedures were performed during the light phase of the light/dark cycle.

### Experimental apparatus

The PRL tests were conducted in operant conditioning chambers (Med Associates; St Albans, Vermont, USA) enclosed within a sound-attenuating box. Each chamber was equipped with a fan, house light, speaker, a food dispenser set to deliver a sucrose pellet (Dustless Precision Pellets, 45 mg; Bio-Serv, New Jersey, USA), fluid receptacle, and two retractable levers located at the sides of the feeder.

Tests examining alcohol-seeking and taking behaviours were conducted in the same operant chambers, except that the levers were located on the wall opposite to the liquid dispenser, to create a new experimental environment that would not interfere with habits the animals acquired during sensitivity screening.

### Experimental schedule

**Fig. 1:**
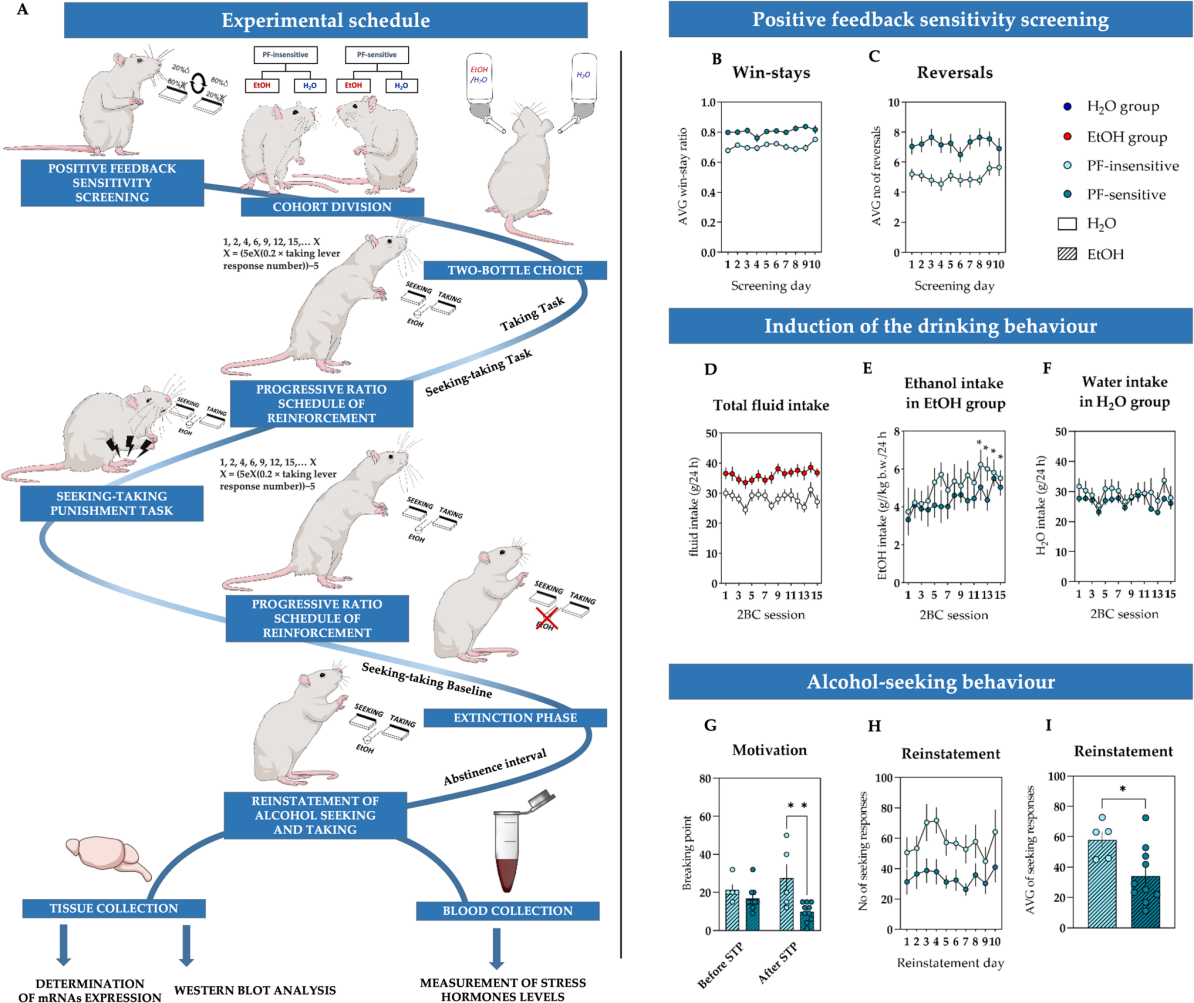
The experimental schedule and parameters measured in animals classified as insensitive and sensitive to positive feedback (PF) and in water- (H_2_O) and alcohol-drinking (EtOH) groups during feedback sensitivity screening, intermittent access two-bottle choice (2BC) sessions, and tests examining alcohol-seeking and taking behaviours. **A** To determine the effects of insensitivity/sensitivity to PF on the transition from controlled use to uncontrollable alcohol abuse, a cohort of rats was trained and tested in a series of PRL tests, and based on this “PF sensitivity screening”, each rat was classified as insensitive or sensitive to PF. The cohort was further divided into alcohol (EtOH) and water (H_2_O) drinking groups. To induce alcohol drinking behaviour and to measure progression in the amount of consumed alcohol, the rats were tested in the 2BC intermittent access paradigm. Subsequently, following the initial training in the taking and seeking-taking (ST) tasks, the rats’ motivation to seek alcohol was measured using the progressive ratio schedule of the reinforcement (PRSR) paradigm. In the next steps, the influence of insensitivity/sensitivity to PF on alcohol-seeking behaviour was measured in the instrumental seeking-taking punishment (STP) task, following which the animals’ motivation to seek alcohol was evaluated again using PRSR. Following rebaseline measurements of the seeking-taking behaviour, the effects of trait insensitivity/sensitivity to PF on alcohol-seeking behaviour were evaluated following termination of alcohol availability (extinction phase) and following 1 month of abstinence (reinstatement). At the end of the experiment, the animals were sacrificed, and the effects of prolonged alcohol consumption and its withdrawal on gene expression, protein levels, and blood levels of stress hormones were compared between PF-insensitive and PF-sensitive animals. **B** Average proportion of win-stay behaviours following a reward in rats classified as PF-insensitive (light blue circles) and PF-sensitive (dark blue circles) across all 10 screening Probabilistic Reversal Learning (PRL) tests; **C** Average number of reversals made by animals classified as PF-insensitive (light blue circles) and PF-sensitive (dark blue circles) during the 10 screening PRL tests; **D** Average daily fluid intake during all 15 2BC sessions in H_2_O (white circles) and EtOH (red circles) groups; **E** Average alcohol intake (g/kg b.w./24 h) during all 15 2BC sessions in PF-insensitive (light blue circles) and PF-sensitive (dark blue circles) rats from the EtOH group. An asterisk indicates a significant (*p* < 0.05) difference in average (for all rats in the EtOH group) alcohol consumption on a given 2BC session compared to the first 2BC session; **F** Average water intake (g/24 h) during all 15 2BC sessions in PF-insensitive (light blue circles) and PF-sensitive (dark blue circles) rats from H_2_O group; **G** the effects of PF sensitivity on motivation to seek alcohol. The break point in the PRSR tests conducted before and after the STP sessions in rats classified as PF-insensitive (light blue dashed bars) and PF-sensitive (dark blue dashed bars). A double asterisk indicates a significant (*p* < 0.01) difference between the PF-insensitive and PF-sensitive groups; **H**, **I** daily and averaged number of seeking responses during 10 ST tests following a 30-day abstinence interval. A single asterisk indicates a significant (*p* < 0.05) difference between the PF-insensitive and PF-sensitive groups. Data are presented as the mean ± SEM

The experimental schedule is summarized in Fig. 1A.

### Measuring sensitivity to PF using the PRL test

#### Initial training

During the initial stage of training, one of the levers (left/right levers counterbalanced) was extended. Each press on this lever resulted in sugar pellet delivery, with a fixed ratio 1 (FR1) schedule of reinforcement. After each press, the lever retracted for 3 s (inter-trial interval (ITI)) before the next trial began. If the lever was not pressed within 10 s, it was considered an omission. The criterion of less than 20% omissions had to be met before progressing to the second stage of the training. There was no pre-determined limit on the number of trials, and each training session lasted for 30 min.

The second stage of training involved random presentations of either the left or right lever. The rats were required to press each lever at least 30 times within 30-min training session. To avoid side bias during the PRL task, animals had to respond with similar frequency on both levers. This was achieved by ensuring that they made less than 7.5% omissions on each lever (i.e., less than 15% total omissions but equally distributed between the levers) for 3 consecutive training days. Once this criterion was met, the animals were ready to be tested in the PRL procedure.

#### PRL training

Each training session consisted of 200 trials, of which each lasted for a maximum of 22 s. The start of a trial was signalled by the house light, which remained on until the end of the trial. Two seconds after the trial started, both levers were presented, and one of them was randomly assigned as the “correct” lever, which delivered a reward (one sucrose pellet) 80% of the times it was pressed. Pressing on this lever was followed by 5 s ITI. A press on the other, “incorrect” lever would result in a rewarding outcome only 20% of the times it was pressed. A failure to respond within 10 s triggered the 5 s ITI and was counted as an omission. During the ITI, both levers were retracted, and the house light was turned off. The same ITI directly followed a punishing outcome i.e. no reward on 20% of the “correct” and 80% of the “incorrect” lever presses. The use of probabilistic reinforcement increased the complexity of the task in such a way that the information from any given choice was insufficient to guide future behaviour, and subjects must engage cognitive functions to track the reward history for both stimuli to determine the stimulus that was more beneficial overall.

After every 8 consecutive “correct” lever presses (regardless of the outcome), the criterion for the reversal of the outcome probabilities was reached. The previously “correct” lever now became “incorrect” and vice versa. This pattern was followed until the end of the session. The PRL training phase was repeated daily until the individual animals achieved sufficient performance levels. The criteria to be met were a minimum of 3 reversals completed during 3 consecutive training sessions, with less than 15% omissions per session.

#### Parameters measured in the PRL test

To measure rats’ sensitivity to PF, all rewarded outcomes (true and misleading) followed by a decision to stay with the lever that delivered them (win-stays) were counted jointly for the “correct” and “incorrect” levers and expressed as a ratio of all rewarded outcomes on that lever. Additionally, the number of reversals completed during the experimental session served as a measure of the general performance of the animals on the task and as a measure of cognitive flexibility.

#### PF sensitivity screening

After achieving a stable performance in the PRL (a minimum of 3 reversals and less than 15% omissions in three consecutive sessions), the rats were tested in 10 consecutive PRL tests conducted over 10 consecutive days. Based on this “PF sensitivity screening”, the animals were divided into PF-insensitive and PF-sensitive groups using a median split. The division was made based on the average ratio of pressing the same lever (win-stays) following both true and misleading rewards across all 10 screening tests.

### Intermittent access to alcohol in the 2BC paradigm

To induce drinking behaviour and to determine the level of alcohol consumption in the EtOH group, 15 sessions of the 2BC procedure were conducted every second day. During the 2BC tests, animals were separated into individual cages for 24 h, where they were presented with either one bottle of 10% EtOH (w/w) and one bottle of water or two bottles of water for the EtOH and H_2_O groups, respectively. To avoid potential effects of side preferences in drinking, the position of the bottles was changed after 12 h. The bottles were weighed before and after each session to determine total fluid intake for both groups (g/24 h) and alcohol consumption in the EtOH group (g EtOH/kg of body weight (b.w.)/24 h). The volume of liquids consumed was calculated as the difference in bottle weights at the beginning and the end of each session, subtracting the volume lost due to dripping from bottles on an empty cage.

### Acquisition of alcohol-seeking behaviour: taking task

The rats were trained to associate the pressing of the taking lever with the alcohol or water (in the case of water drinking control groups) delivery under a FR1 schedule of reinforcement. Each trial started with the insertion of the randomly assigned taking lever and a house light on (left/right levers counterbalanced). Pressing on the lever resulted in dipper presentation on the opposite side of the box, delivery of 0.1 ml of 15% EtOH (w/w) or water for the EtOH and H_2_O groups, respectively, and simultaneous retraction of the taking lever. No response in 10 s triggered the 10 s ITI and was counted as an omission. Regardless of the result, each trial was followed by an ITI during which the levers were retracted and alcohol/water was not available. Rats were limited to a maximum of 60 rewards for a 30-min training session. After achieving the performance criterion of a minimum of 20 taking responses in three consecutive sessions, the animals were shifted to the ST task.

### Acquisition of alcohol-seeking behaviour: ST task

During this task, each trial started with the insertion of the seeking lever, opposite to the randomly assigned taking lever, which remained retracted. The seeking lever response started the randomly applied interval of 1 to 15 s (RI 1–15 s), after which the taking lever was extended. Pressing of the taking lever under FR1 resulted in the presentation of the dipper on the opposite side of the box, delivery of 0.1 ml of 15% EtOH (w/w), and simultaneous retraction of both levers. Each trial was followed by a 10-s ITI during which both levers were retracted and alcohol was not available. If the animal did not press the seeking lever, the lever remained extended until the end of the session. Rats were limited to a maximum of 100 rewards for 45 min sessions. When animals performed a minimum of 20 responses in 3 consecutive sessions, their motivation to seek alcohol was tested in the PRSR task.

### Measuring motivation to seek alcohol using the PRSR

Each trial started with the extension of the seeking lever, pressing on which resulted in taking lever extension after RI 1–15 s. The number of seeking lever presses required to produce this effect increased progressively with each successive taking lever response and EtOH delivery. The steps of the exponential progression used in our study were the same as those previously developed by Roberts and Bennet (Roberts and Bennett 1993) and previously used by Rygula and colleagues (Rygula et al. 2015b) and were based on the following equation: response ratio = (5eX(0.2 × taking lever response number)) − 5, rounded to the nearest integer. Thus, the values of the steps were 1, 2, 4, 6, 9, 12, 15, 20, 25, 32, 40, 50, 62, 77, 95, 118, 145, 178, 219, 268, 328, 402, 492, 603, etc. Each trial was followed by 10 s ITI when both levers were retracted. Sessions lasted 30 min. The maximum number of lever presses a subject was willing to exert to obtain a reward was referred to as break point and served as the measure of motivation for alcohol.

### Acquisition of alcohol-seeking behaviour: STP task

After the PRSR test, we measured the persistence of seeking behaviour in the face of aversive consequences using the STP task. In this paradigm, each trial started as described for the ST task, with the insertion of the seeking lever. Seeking lever response resulted either in a 1 s electric shock (0.10–0.50 mA), administered through a grid floor, or the extension of the taking lever after a random interval (RI 1–15 s). During each session, rats were limited to a maximum of 25 trials, of which 17 (70%) were reinforced by EtOH delivery following the lever response, and 8 (30%) were punished with foot shock. The intensity of the shock increased in daily sessions according to the following pattern: 0.10, 0.20, 0.30, 0.30, 0.40, 0.40, 0.50, and 0.50 mA. Although punishment occurred randomly in each session, never more than two consecutive trials resulted in a foot shock, and the first trial of the session was always reinforced. Upon completion of the STP task, rats were challenged again in the PRSR test and rebaselined in 5 ST test sessions.

### Extinction and reinstatement of alcohol-seeking and taking behaviours

After the rebaseline procedure, all animals underwent between 5 and 20 (AVG = 12) daily 15 min extinction sessions, during which the seeking lever response under RI 1–15 s resulted in taking lever extension; however, the taking lever presses had no programmed consequences, and alcohol was not available. After reaching the extinction criterion (less than 5 seeking responses in 3 consecutive sessions), the rats were alcohol deprived and not tested for the following 30 days. This abstinence interval was chosen to more naturally reflect condition of relapse in humans after a longer period of time (Moe et al. 2022).

After 30 days of abstinence, the rats underwent a series of ST tests to measure how quickly they reinstate their alcohol-seeking behaviour and bring their performance up to the basal level. The animals were tested until they reached the criterion of an average number of seeking responses from 5 tests equal to or higher than the average number of seeking responses from the 5 rebaseline ST tests.

### Tissue collection

At the end of the behavioural part of the experiment, the sacrificed rats were decapitated, and 5 brain structures were collected for biochemical analyses: 3 cortical (medial prefrontal cortex (mPFC), anterior cingulate (ACC), and orbitofrontal cortex (OFC)) and 2 subcortical areas (nucleus accumbens (Nacc) and amygdala (Amy)). All the above-mentioned brain areas have been previously demonstrated to be involved in the mediation of sensitivity to feedback (Clarke et al. 2014; Cools et al. 2002, 2009; Cservenka 2016; Dalton et al. 2014; Golebiowska and Rygula 2017b). Tissue was taken based on the “Rat Brain Atlas” of Paxinos & Watson (Paxinos and Watson 1998) and according to Achterberg and colleagues (Achterberg et al. 2015). The structures were frozen on dry ice and stored at − 70° C for further analysis.

### Gene selection

The effects of trait sensitivity to PF and alcohol drinking on gene expression within selected brain regions were assessed using TaqMan Low Density Arrays (TLDA, described below). The predesigned TLDA allowed for the screening of 32 genes (29 candidate genes, 2 reference genes, and 1 endogenous gene control), which were potentially involved in mediating the effects of PF on alcohol-seeking and drinking in rats. Based on an extensive literature search and analysis of the effects of various genetic and pharmacological manipulations on sensitivity to feedback, 4 groups of genes were chosen. (1) Genes involved in the functioning and regulation of the serotonin (5-HT) system (e.g., serotonin receptors: 5-HT1A, 5-HT2A, serotonin transporter (SERT) and tryptophan hydroxylase). Indeed, it has been demonstrated in humans (Chamberlain et al. 2006; Cools et al. 2008; den Ouden Hanneke et al. 2013), nonhuman primates (Rygula et al. 2015a) and rodents (Bari et al. 2010; Golebiowska and Rygula 2017a; Ineichen et al. 2012; Rygula et al. 2014) that acute and permanent manipulations of the activity of the 5-HT system affect sensitivity to feedback. (2) Because, along with 5-HT, dopamine (DA) is the second neurotransmitter critically implicated in learning from feedback (Cools et al. 2009; Klein et al. 2007; Pessiglione et al. 2006), the second group of screened genes was chosen among those involved in dopaminergic neurotransmission (e.g., dopamine receptors: D1, D2, D4, dopamine transporter (DAT), tyrosine hydroxylase, monoaminooxidase (MAO) A and B, and catechol-O-methyltransferase (COMT)). (3) Because changes in brain DA neurotransmission often result from secondary neuroadaptations in other neurotransmitter systems, such as glutamate (Kauer and Malenka 2007) and γ-aminobutyiric acid (GABA) (Volkow et al. 2010), genes associated with these 2 neurotransmitter systems, e.g., the ionotropic glutamate receptors NMDA and AMPA, the metabotropic glutamate receptors mGLU2, mGLU3, and mGLU5, glutamate decarboxylase (GAD), and GABAA and GABAB receptors, constituted the third analyzed group. (4) The fourth group included genes involved in EtOH metabolism, such as catalase and alcohol dehydrogenase (Hipolito et al. 2007). (5) Last but not least, ribosomal protein L32 (*Rpl32*) and peptidylprolyl isomerase A (*Ppia*) were used as reference genes as described previously (Gąska et al. 2012).

### Isolation of RNA from the brain structures

Total RNA was isolated from collected tissues using the RNeasy Plus Mini Kit (Qiagen, Germantown, MD, US) according to the manufacturer’s instructions. The samples (8–11 per group) were homogenized with 600 µl of RTL Plus buffer with β-mercaptoethanol for 4 min at 50 Hz with TissueLyser LT (Qiagen, Germantown, MD, US). Then, gDNA Eliminator spin columns were used. Then, 600 µl of 70% ethanol was added to each sample and transferred to the RNeasy spin column. After washing the column, 30 µl of RNase-free water was added to the column for RNA elution. The quality and quantity of the isolated total RNA were evaluated by a NanoDrop ND-1000 (Thermo Fisher Scientific) and an Experion microcapillary electrophoresis system (Bio-Rad, Hercules, California, USA). Samples that passed the quality threshold (RIN > 8.0) were used for further experiments.

### Isolation of protein from the brain structures

During RNA isolation, the protein was obtained using the cold acetone precipitation method. For this, 800 µl of cold acetone was added to 100 µl of flow-through acquired after RNA binding to the RNeasy spin column. The protein was precipitated for 1.5 h at -20 °C and centrifuged for 15 min at 14,000 rpm at 4 °C. The pellet was dissolved in a buffer containing 7 M urea, 2 M thiourea, 40 mM Tris, 4% CHAPS, 65 mM DTT, and protease inhibitor cocktail (Thermo Fisher Scientific) and stored at − 20 °C for future analysis.

### Determination of mRNA expression by TaqMan gene expression array cards

The isolated RNAs were used to synthesize cDNA transcripts according to the manufacturer’s protocol of the High-Capacity cDNA Reverse Transcription Kit (Thermo Fisher Scientific). The amount of RNA was equalized for all samples depending on the structure. The obtained cDNA was mixed with TaqMan Universal PCR Master Mix, No AmpErase UNG (Thermo Fisher Scientific) to perform the RT-qPCRs. qPCRs were carried out simultaneously using Custom TaqMan Gene Expression Array Cards (Thermo Fisher Scientific). The 29 genes that are potentially involved in the mediation of the effects of feedback sensitivity and alcohol-seeking and drinking in rats were placed on one Array Card. One Array Card was used to examine the mRNA expression of four samples in triplicate. The RT-qPCRs were run on a QuantStudio 12 K Flex System (Applied Biosystems, Waltham, Massachusetts, US). Data were further analysed with QuantStudio 12 K Flex Software (Applied Biosystems). A Ct value above 34 was considered undetectable. The same threshold equal to 0.20 was set for all samples for comparison. Then, the data were analysed with qBasePLUS 3.1 software (Biogazelle, Zwijnaarde, Belgium) (Hellemans et al. 2007), which uses a generalized model of the delta-delta-Ct approach, thereby supporting the use of gene specific amplification efficiencies and normalization with multiple reference genes. Rpl32 and Ppia were selected for normalization.

### Western blot analysis

The concentration of proteins was determined using the Bradford Reagent (Sigma‒Aldrich, Saint Louis, MO, USA) following the manufacturer’s protocol. Equal concentrations of proteins were mixed with 4X Bolt® LDS Sample Buffer (Invitrogen, Waltham, MA, USA) and 10X Bolt® Sample Reducing Agent (Invitrogen) and then denatured at 70 °C for 10 min. Samples were separated on Bolt™ 4–12% Bis–Tris Plus Gels (Invitrogen) under reducing conditions in 20X Bolt® MES SDS Running Buffer (Invitrogen), incubated in 20% ethanol for 10 min, and transferred to immunoblot nitrocellulose membranes (iBlot® 2 Transfer Stacks, nitrocellulose, Invitrogen, Waltham, MA, USA) in accordance with the manufacturer’s protocol. Primary and secondary antibodies were suspended in an iBind™ Solution Kit followed by membrane incubation on iBind™ Cards using the iBind™ Western Device (SLF1000, Invitrogen, Waltham, MA, USA) for 2.5 h or overnight. The following concentrations of primary antibodies were used to determine protein levels: 1:200 for MAO-B (mouse, cat. number: sc-515354; Santa Cruz Biotechnology), 1:2000 for ADH1 (rabbit, cat. number PA5- 8730, Invitrogen), 1:1000 for DRD1 (rat, cat. number: D2944, Sigma‒Aldrich), 1:50 for GABABR2 (mouse, cat. number: sc-393286, Santa Cruz Biotechnology), 1:2000 for 5-HT1A (rabbit, cat number: PA5-77,745 Invitrogen), 1:1000 for 5-HT2A (rabbit, cat. number: ab216959, Abcam), 1:1000 for 5-HT3A (rabbit, cat. number: bs-2126R Bioss antibodies), 1:200 for CAT (mouse, cat. number: sc-271803) and 1:2000 for SERT (rabbit, cat. number: PA5-80,032, Invitrogen). The secondary anti-mouse (cat. number: A9044, Sigma Aldrich) and anti-rabbit (cat. number: ab6721, Abcam) antibodies were used at concentrations of 1:20 000. Anti-rat antibodies were used at a concentration of 1:1000 (cat. number: HAF005, Biotechne). As a loading control, β-actin (monoclonal anti-β-actin antibody produced in mouse, A5441, Sigma‒Aldrich, Saint Louis, MO, USA) was applied at a concentration of 1:20 000, and its corresponding secondary antibody (anti-mouse IgG, A9044, Sigma‒Aldrich, Saint Louis, MO, USA) was applied at a concentration of 1:20 000. The electrophoretic bands were detected using the Clarity™ Western ECL Substrate (Bio-Rad, Hercules, CA, USA) and FUJIFILM LAS-4000 (Fujifilm Life Science, USA) device. Blot analysis was performed using ImageJ 1.53e software (Wayne Rusband and NIH, USA). Due to limited gel spots, a minimum of three samples from different groups were included in each blot.

### Measurement of blood stress hormone levels

To assess whether trait sensitivity to PF interacts with the effects of prolonged alcohol consumption on the level of stress hormones, on the day after the last behavioural procedure (between 09:00 am and 12:00 pm), the rats from the EtOH and H_2_O groups were sacrificed and tested for blood concentrations of adrenocorticotropic hormone (ACTH) and corticosterone (CORT) using a Merck Rat Stress Hormone Magnetic Bead Panel. For all animals, the blood was collected, after clotting, centrifuged at 1500 × g at 4 °C for 10 min. The obtained serum was stored at − 80 °C and analysed for ACTH and corticosterone concentrations according to the manufacturer’s instructions.

### Statistics

The data were analysed using SPSS (version 25.0, SPSS Inc., Chicago, IL, USA). The normality of the sensitivity to feedback data was verified using the Kolmogorov–Smirnov test. Nonparametric data were normalized by square rooting and, where appropriate, removing outliers. The physiological and molecular data were analysed using 2-way ANOVA. When the data could not be normalized, the Kruskall Wallis test was used. The screening, 2BC, ST, STP, extinction, and reinstatement data were analysed using two-way repeated-measures ANOVAs with the within-subject factor of test day/session and the between-subject factor of sensitivity to PF.

The differences between the PF-insensitive and PF-sensitive groups of rats in the average quantity of alcohol consumed and the number of tests needed to achieve extinction and reinstatement criteria were analysed using t tests or, for nonparametric data, using Mann– Whitney *U* tests. For pairwise comparisons, we adjusted the values using Sidak’s correction for multiple comparisons (Howell 1997). All tests of significance were performed at *α* = 0.05. We tested the homogeneity of variance using Levene’s test, and for repeated-measures analyses, we confirmed sphericity using Mauchly’s test. The data are presented as the mean ± SEM.

## Results

### PRL training and testing

All animals fulfilled the PRL training criteria and qualified for PRL screening. On average, the animals reached the criteria after 6.78 ± 0.42 PRL tests. The PF-insensitive/PF-sensitive rats did not differ significantly in the number of PRL tests needed to reach the criterion (Mann‒ Whitney: *p* = 0.631, Figure S1).

### PF sensitivity screening

The average proportion of win-stay behaviours in the animals classified as PF-insensitive (*N* = 20) ranged from 0.662 to 0.738, with an average of 0.706 ± 0.005. The average proportion of win-stay behaviours in the animals classified as PF-sensitive (*N* = 20) ranged from 0.745 to 0.892, with an average of 0.806 ± 0.011. The difference in sensitivity to PF between both subgroups (*F*_(1,38)_ = 70.90, *p* < 0.001) was stable across the screening period (not significant effect of screening day (*F*_(9,342)_ = 1.369, *p* = 0.201) and not significant sensitivity × screening day interaction (*F*_(9,342)_ = 1.021, *p* = 0.422)). The average number of reversals made by the animals classified as PF-insensitive was significantly lower than that for animals classified as PF-sensitive (*F*_(1,38)_ = 35.800, *p* < 0.001). This difference in the reversal performance between both subgroups was stable across the screening period (not significant effect of screening day (*F*_(9,342)_ = 0.555, *p* = 0.833) and not significant sensitivity × screening day interaction (*F*_(9,342)_ = 0.617, *p* = 0.782)). Individual data (proportion of win-stay and reversal performance across all 10 screening PRL tests) of all 40 animals are presented in figures S2A and S2B respectively.

As only 15 out of the 20 EtOH rats achieved the criteria of taking and ST tests (described in the next sections), and 19 out of the 20 H2O rats (because of the mistake in the treatment) were analysed further, the screening data for these 34 animals were as follows:

The average proportion of win-stay behaviours in the animals classified as PF-insensitive (*N* = 16) ranged from 0.662 to 0.738, with an average of 0.705 ± 0.007. The average proportion of win- stay behaviours in the animals classified as PF-sensitive (*N* = 18) ranged from 0.745 to 0.892, with an average of 0.805 ± 0.012. The difference in sensitivity to PF between both subgroups (*F*_(1,32)_ = 51.61, *p* < 0.001; Fig. 1B) was stable across the screening period (not significant effect of screening day (*F*_(9,288)_ = 1.449, *p* = 0.167) and not significant sensitivity × screening day interaction (*F*_(9,288)_ = 1.188, *p* = 0.302)).

The average number of reversals made by the animals classified as PF-insensitive was significantly lower than that for animals classified as PF-sensitive (*F*_(1,32)_ = 27.27, *p* < 0.001; Fig. 1C). This difference in the reversal performance between both subgroups was stable across the screening period (not significant effect of screening day (*F*_(9,288)_ = 0.494, *p* = 0.878) and not significant sensitivity × screening day interaction (*F*_(9,288)_ = 0.778, *p* = 0.637)).

### Cohort division

Based on PF sensitivity screening, the animals were classified into two groups: PF-insensitive (*N* = 20) and PF-sensitive (*N* = 20). Then, according to the applied treatment, they were further randomly divided into four subgroups: EtOH_PF-insensitive_ (*N* = 9), EtOH_PF-sensitive_ (*N* = 11), H_2_O_PF-insensitive_ (*N* = 11), and H_2_O_PF-sensitive_ (*N* = 9) animals.

Because, as mentioned above, only 34 out of 40 initially trained animals completed all experimental procedures, ultimately, the groups analyzed in the present experiment were as follows: EtOH_PF-insensitive_ (*N* = 5), EtOH_PF-sensitive_ (*N* = 10), H2O_PF-insensitive_ (*N* = 11), and H2O_PF-sensitive_ (*N* = 8) animals.

### Induction of drinking behaviour

During the 15 2BC sessions, rats from the EtOH group consumed more fluids than their conspecifics from the H_2_O group (significant main effect of treatment (*F*_(1, 32)_ = 11.000, *p* = 0.002); Fig. 1D). Moreover, they significantly (*p* < 0.05) increased their alcohol intake with an average from the first test of 3.47 ± 0.58, reaching an average of 5.20 ± 0.32 g/kg/24 h in the last session (significant main effect of session (*F*_(14, 182)_ = 2.613, *p* = 0.002, Fig. 1E). We did not observe significant differences in alcohol consumption between PF-insensitive/PF-sensitive animals (nonsignificant effect of sensitivity (*F*_(1, 13)_ = 0.103, *p* = 0.329), nonsignificant session × sensitivity interaction (*F*_(14, 182)_ = 0.456, *p* = 0.953, Fig. 1E). There was no significant difference in water consumption between PF-insensitive/PF-sensitive rats from the H_2_O group (nonsignificant sensitivity effect (*F*_(1, 17)_ = 0.573, *p* = 0.460; no sensitivity × session interaction (*F*_(14, 238)_ = 0.805, *p* = 0.663, Fig. 1F)).

One rat from the control group (PF-sensitive) was removed from the analysis and further experiments due to a mistake in the applied treatment. As only 15 out of the 20 rats achieved the criteria of taking and ST tests described in the next section, only these 15 animals were analysed regarding their consumption of alcohol in 2BC sessions and subsequent experimental steps.

### Acquisition of alcohol-seeking behaviour in rats

In the next step, the animals from the EtOH and H_2_O groups were trained to associate the pressing of the taking lever with the alcohol or water delivery under FR1. As mentioned above, only 15 out of the 20 rats from the EtOH group achieved the criteria for taking and ST tests. None of the rats from the H_2_O group met the criteria.

After reaching the ST criterion, the rats were tested in the STP task. As the shock intensity increased from 0.10 to 0.50 mA during consecutive sessions, all rats gradually decreased the number of trials completed compared to the initial session (main shock intensity effect (*F*_(7,91)_ = 5.990, *p* < 0.001, Figure S3A). We did not observe significant differences in the number of trials completed between the PF-insensitive and PF-sensitive groups of rats (nonsignificant effect of sensitivity to PF (*F*_(1, 13)_ = 0.011, *p* = 0.919) and nonsignificant sensitivity to PF × shock intensity interaction (*F*_(8, 104)_ = 0.471, *p* = 0.853)).

### Motivation to seek alcohol before and after the introduction of punishment

Additionally, to measure the impact of punishment in the STP task on rats’ motivation for alcohol-seeking, we conducted two PRSR tests. In the first one, executed before STP tests, the rats’ average break point was 18.33 ± 1.66, while in the second one, performed after the STP test, the average break point was 15.67 ± 3.36.

PF sensitivity had no significant effect on the break point of rats tested before the STP. Interestingly, however, the animals classified as PF-insensitive reached a significantly higher break point during the PRSR test performed after the STP tests than their PF-sensitive conspecifics (significant sensitivity effect (*F*_(1, 13)_ = 8.532, *p* = 0.012) and significant sensitivity × test interaction (*F*_(1, 13)_ = 6.185, *p* = 0.027); Fig. 1G).

### Extinction and reinstatement of alcohol-seeking behaviour

After the second PRSR test, all animals underwent 5 additional ST tests. Following the rebaseline, rats were tested under ST extinction conditions, during which alcohol was not available. The number of sessions needed to achieve the extinction criterion ranged from 5 to 20, with an average of 12.07 ± 1.29. Sensitivity to PF had no significant impact on the length of extinction (t test; p = 0.808, Figure S3B).

The effects of PF sensitivity on the reinstatement of alcohol-seeking were assessed following 30 days of forced abstinence. Throughout 10 tests, most of the animals reinstated their preextinction level of seeking responses. PF-sensitive animals showed a significantly lower number of seeking responses, with an average of 34.18 ± 6.00, than PF-insensitive rats, with an average of 57.96 ± 5.44 (main effect of sensitivity (*F*_(1, 13)_ = 6.400, *p* = 0.025, Fig. 1H, I) and a nonsignificant sensitivity × test interaction (*F*_(9, 117)_ = 0.403, *p* = 0.931). There was no significant difference in the number of tests needed to achieve the criterion between the PF-insensitive and PF-sensitive groups (Mann‒Whitney test, *p* = 0.445). Two animals (PF-sensitive) did not meet the reinstatement criterion.

### The effects of PF sensitivity and alcohol consumption on gene expression levels

**Fig. 2:**
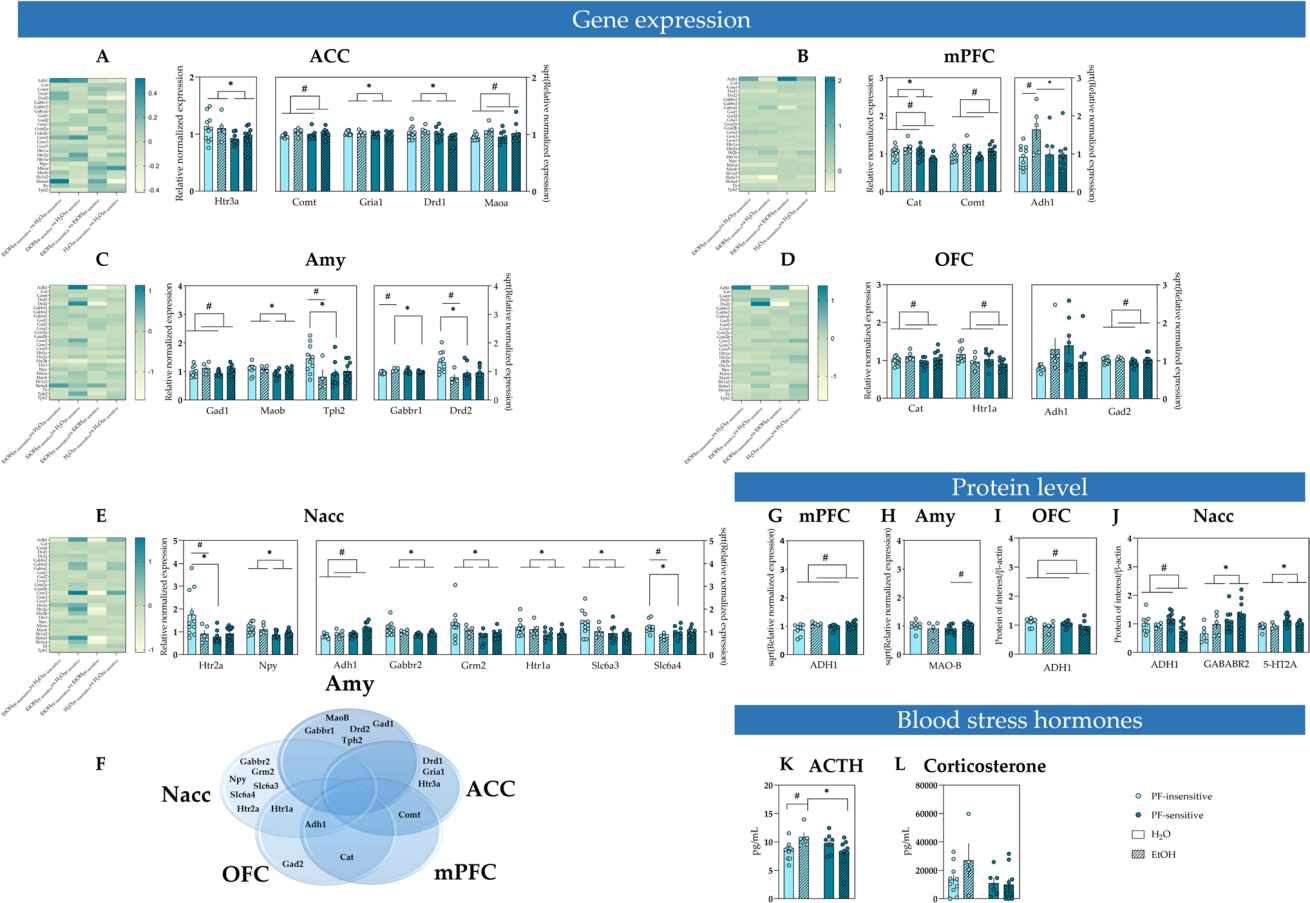
Molecular and physiological differences associated with high and low sensitivity to positive feedback (PF) and alcohol drinking in rats. **A**–**E** Heatmaps and bar graphs demonstrating statistically significant differences in the relative normalized expression of the genes of interest in PF-insensitive (light blue bars) and PF-sensitive animals (dark blue bars) belonging to H_2_O (open bars) and EtOH (dashed bars) drinking groups in **A** anterior cingulate cortex (ACC), **B** medial prefrontal cortex (mPFC), **C** amygdala (Amy), **D** orbitofrontal cortex (OFC), and **E** nucleus accumbens (Nacc). A single asterisk indicates significant (*p* < 0.05) difference between PF-insensitive and PF-sensitive groups. # indicates a significant (*p* < 0.05) difference between the EtOH and H_2_O groups. **F** Venn diagram illustrating genes altered by sensitivity to PF and/or treatment in the brain structures studied using a TaqMan Array Card. **G**–**J** Protein to β-actin ratio for proteins selected based on the gene expression analysis in PF-insensitive (light blue bars) and PF-sensitive animals (dark blue bars) belonging to H_2_O (open bars) and EtOH (dashed bars) groups in **G** mPFC, **H** Amy, **I** OFC, and **J** Nacc; A single asterisk indicates significant (*p* < 0.05) difference between PF-insensitive and PF-sensitive groups. A number sign indicates significant (*p* < 0.05) difference between PF-sensitive animals belonging to H_2_O and EtOH groups. K) Blood ACTH concentration in PF-insensitive (light blue bars) and PF-sensitive (dark blue bars) animals in EtOH (dashed bars), and H_2_O groups (open bars). A single asterisk indicates a significant (*p* < 0.05) difference between EtOH and H_2_O groups. A number sign indicates a significant (*p* < 0.05) difference between the PF-insensitive and PF-sensitive groups. L) Blood corticosterone concentration in PF-insensitive (light blue bars) and PF-sensitive animals (dark blue bars) in the EtOH (dashed bars) and H_2_O groups (open bars). Data are presented as the mean ± SEM

Statistical analysis of the effects of trait sensitivity to PF on the expression of genes revealed statistically significant intergroup differences in all investigated regions of interest except the OFC. In the ACC, the mRNA level was higher in the PF-insensitive rats compared to their PF-sensitive conspecifics, for *Drd1* (*F*_(1, 29)_ = 4.556, *p* = 0.041), *Gria1* (*F*_(1, 30)_ = 4.809, *p* = 0.036), and *Htr3a* (*F*_(1, 30)_ = 5.855, *p* = 0.022) (Fig. 2A); in the mPFC for *Cat* (*F*_(1, 30)_ = 9.431, *p* = 0.005) (Fig. 2B) and in the Amy for *Maob* (*F*_(1, 28)_ = 5.804, *p* = 0.023) (Fig. 2C). In the Nacc, the mRNA level was higher in PF-insensitive animals for *Gabbr2* (*F*_(1, 29)_ = 6.557, p = 0.016), *Grm2* (*F*_(1, 29)_ = 4.863, *p* = 0.036), *Htr1a* (*F*_(1, 30)_ = 6.452, *p* = 0.017), *Htr2a* (*F*_(1, 30)_ = 4.367, *p* = 0.045), *Npy* (*F*_(1, 30)_ = 10.02, *p* = 0.004), and *Slc6a3* (*F*_(1, 30)_ = 5.166, *p* = 0.030) (Fig. 2E).

The analysis also revealed significant interactions between the effects of trait sensitivity to PF and alcohol drinking on the expression of *Adh1* (*F* (1, 29) = 5.048, *p* = 0.032) in mPFC and *Gabbr1* (*F*_(1, 27)_ = 9.466, *p* = 0.005) in the Amy with mRNA level higher in EtOH_PF-insensitive_ group compared to H_2_O_PF-insensitive_ and to EtOH_PF-sensitive_ groups (Fig. 2B, C). For *Tph2* (*F* _(1, 28)_ = 4.732, *p* = 0.038), and *Drd2* (*F*_(1, 28)_ = 6.200, *p* = 0.019) in the Amy, and for *Htr2a*, (*F*_(1, 30)_ = 4.606, *p* = 0.040), and *Slc6a4* (*F*_(1, 29)_ = 4.977, *p* = 0.034) in the Nacc,the mRNA level was higher in H_2_O_PF-insensitive_ animals compared to H_2_O_PF-sensitive_ and EtOH_PF-insensitive_ groups (Fig. 2C, E). We also revealed significant intergroup differences in the *Adh1* expression within the OFC (Kruskal‒Wallis test: *p* = 0.038).

The significant effects of alcohol drinking (treatment) were observed, with mRNA level higher in the EtOH group compared to H_2_O-drinking ones, for *Comt* (F _(1, 30)_ = 5.223, *p* = 0.030) and *Maoa* (*F*_(1, 29)_ = 4.732, *p* = 0.038) in the ACC (Fig. 2A), for *Adh1* (*F*_(1, 29)_ = 5.072, *p* = 0.032), *Cat* (*F*_(1, 30)_ = 7.312, *p* = 0.011) and *Comt* (*F*_(1, 30)_ = 18.320, *p* < 0.001) in the mPFC (Fig. 2B), for *Gad1* (*F*_(1, 28)_ = 4.338, *p* = 0.047) and *Drd2* (*F*_(1, 28)_ = 5.092, *p* = 0.032) in the *Amy* (Fig. 2C), for *Cat* (*F*_(1, 30)_ = 5.351, *p* = 0.028), *Gad2* (*F*_(1, 30)_ = 6.329, *p* = 0.018) and *Htr1a* (*F*_(1, 30)_ = 6.362, *p* = 0.017) in the OFC (Fig. 2D), and for *Adh1* (*F*_(1, 27)_ = 8.590, *p* = 0.007) in the Nacc (Fig. 2E). For *Slc6a4* (*F*_(1, 29)_ = 6.895, *p* = 0.014) in the Nacc the mRNA level was lower in the EtOH-drinking rats compared to the H_2_O-drinking ones (Fig. 2E).

The results of statistical analysis of the effects of trait sensitivity to PF and its interactions with alcohol drinking on the expression of all investigated genes in all investigated brain regions are demonstrated in Table S1. The genes with significantly different expression in the investigated regions of interest are additionally presented in a Venn diagram (Fig. 2F).

To determine how various RNA expression levels affect protein levels, we performed Western blot analyses on protein products identified by TaqMan Gene Expression Array cards. Statistical analysis of the effects of trait sensitivity to PF on the protein levels revealed statistically significant intergroup differences, with the level of GABA-B receptor subunit 2 (GABABR2, gene: *Gabbr2*) (*F*_(1, 28)_ = 5.422, *p* = 0.027) and serotonin receptor 2A (5-HT2A, gene: *Htr2a*) (*F*_(1, 30)_ = 6.689, *p* = 0.015) higher in PF-sensitive rats compared to PF-insensitive group in the Nacc (Fig. 2J).

The analysis also revealed significant interactions between the effects of trait sensitivity to PF and alcohol drinking on the expression of monoamine oxidase B (MAO-B, gene: *Maob*) (*F*_(1,31)_ = 7.650, *p* = 0.010; Fig. 2H) in the Amy, with protein level higher in the EtOH_PF-sensitive_ compared to H_2_O_PF-sensitive_ group.

The effect of alcohol consumption itself (treatment) was statistically significant for alcohol dehydrogenase 1 (ADH1, gene: *Adh1*) in the mPFC (*F*_(1, 29)_ = 9.059, *p* = 0.005; Fig. 2G), OFC (*F*_(1, 30)_ = 4.753, *p* = 0.037; Fig. 2I) and Nacc (*F*_(1, 30)_ = 7.287, *p* = 0.011; Fig. 2J) with the protein level higher in the EtOH group compared to the H_2_O rats.

The table with the results of statistical analysis of the effects of trait sensitivity to PF and its interactions with alcohol drinking on levels of selected proteins, the expression of which was significantly affected by PF and/or alcohol drinking in all investigated brain regions (Table S2) and original western blot images used for quantification of protein levels are included in Supplemental material.

### The effects of PF sensitivity and alcohol consumption on stress hormone levels

After the reinstatement of alcohol-seeking and taking, all animals were sacrificed and tested for stress hormone levels in the blood. Analysis of the ACTH level data revealed a significant treatment × PF sensitivity interaction (*F*_(1, 29)_ = 9.132, *p* = 0.005) with nonsignificant effects of treatment (*F*_(1, 29)_ = 0.325, *p* = 0.573) and sensitivity (*F*_(1, 29)_ = 1.014, *p* = 0.322; Fig. 2K). The ACTH level in the H_2_O _PF-insensitive_ group was significantly lower than that in the EtOH _PF- insensitive group_ (*p* = 0.044). Additionally, in the EtOH group, the ACTH level in PF-sensitive rats was significantly lower than that in their PF-insensitive conspecifics (*p* = 0.027). There were no statistically significant differences (*p* = 0.237) between the PF-insensitive and PF-sensitive animals in the H_2_O group.

Analysis of the corticosterone level data (Fig. 2L) revealed statistically nonsignificant but observable at the level of statistical trend, higher blood concentrations of corticosterone in PF-insensitive groups of animals compared to their PF-sensitive conspecifics (effect of PF sensitivity *F*_(1, 25)_ = 3.378, *p* = 0.078) regardless of the treatment (nonsignificant effect of treatment (*F*_(1, 25)_ = 1.457, *p* = 0.239) and nonsignificant treatment x sensitivity interaction (F_(1, 25)_ = 1.929, *p* = 0.177).

## Discussion

The results of the present study showed that trait sensitivity to PF in rats determines the level of motivation to seek alcohol following the experience of its negative consequences. Our findings also revealed significant differences between animals classified as insensitive and sensitive to PF in their propensity to reinstate alcohol-seeking behaviours after the period of forced abstinence. The abovementioned effects were accompanied by differences in blood levels of stress hormones and differences in the cortical and subcortical expression of genes and proteins related to dopaminergic, serotonergic, and GABAergic neurotransmission.

Over the past decade, a growing number of studies have demonstrated that the assessment of cognitive correlates of human personality traits in animals could be very useful in searching for potential cognitive biomarkers of various psychiatric disorders. For instance, a study by Rygula and colleagues, using a rodent model, suggested that trait pessimism can serve as a cognitive biomarker of susceptibility to the development of stress-induced anhedonia – a core symptom of depression (Rygula et al. 2013). A few years later, studies by Noworyta and Rygula demonstrated that sensitivity to feedback, measured as a stable and enduring behavioural trait, can determine the effects of acute administration of antidepressant drugs (Noworyta-Sokolowska et al. 2019). In a recent study from our laboratory, Cieslik and colleagues showed that trait sensitivity to negative feedback predicts the vulnerability of rats to the acquisition of compulsive alcohol-seeking and consumption in a situation when these behaviours are being punished (Cieslik et al. 2022). They also showed significant differences between animals classified as less sensitive and more sensitive to negative feedback in their propensity to extinguish alcohol-seeking behaviours after the termination of alcohol availability. The effects of trait sensitivity to PF, described here, are in concert with and complement these recent observations, supporting at the same time the importance of the role that sensitivity to feedback plays in alcohol addiction.

One of the most influential types of classification of variability within AUD in humans is reward and relief drinking, or the extent to which individuals seek alcohol to enhance positive experiences (reward drinking) versus the extent to which individuals seek alcohol to relieve negative emotional and somatic states (relief drinking). Despite promising findings within this domain (linking reward/relief drinking phenotypes with responding to different pharmacological treatments), the lack of preclinical models of reward/relief drinking may hinder efforts to understand these phenomena on neurobiological, molecular and physiological levels. The pattern of results observed in our current study may help to implement such a model that could be based on measuring sensitivity to PF.

Indeed, insensitivity to PF might suggest decreased sensitivity to reward or even anhedonia, reflecting a negative affective state that can be relieved by drinking alcohol. Following this lead, the higher motivation to seek alcohol after the unpleasant and frustrating experience of punishment observed in PF-insensitive rats (Fig. 1G) might also be interpreted as relief drinking. A similar interpretation can be applied to the increased alcohol-seeking observed in the animals insensitive to PF following a stressful and frustrating period of abstinence (Fig. 1H, I). This interpretation of behavioural patterns observed in the animals insensitive to PF is supported by the analysis of stress hormones in the blood, which demonstrated significantly higher level of ACTH (Fig. 2K) and nonsignificantly (statistical trend) elevated corticosterone (Fig. 2L) compared to the PF-sensitive animals, suggesting a higher level of stress in this group. Importantly, elevated level of ACTH was observed only in animals drinking alcohol and were absent in the control group. Additionally, the elevated level of stress hormones in the PF-insensitive animals, which resulted from alcohol-HPA axis interaction, could have also contributed per se to the enhanced motivation to seek and drink alcohol through activation of mesocorticolimbic reward circuitry (Piazza and Le Moal 1997). Indeed, several studies demonstrated that the administration of CORT increases alcohol consumption and adrenalectomy acts in the opposite way (Fahlke and Eriksson 2000; Fahlke et al. 1996).

One could also speculate that the protracted alcohol withdrawal applied in our study resulted in an elevation of reward threshold and increased negative affectivity in animals showing reduced hedonic capacity i.e. insensitivity to PF. Indeed, a stronger behavioural response to forced abstinence demonstrated by the PF-insensitive rats, which was manifested by the higher number of seeking lever presses in the ST task during reinstatement of the instrumental response, seems to support this claim. Since PF-insensitive animals are less sensitive to reward by their nature, alterations in reward threshold and sensitivity caused by prolonged alcohol consumption and withdrawal (Koob et al. 1998; Schulteis et al. 1995) were stronger and more evident in this group. It is worth mentioning that a growing number of studies link hyposensitivity to PF/altered processing of positively valanced information with the symptomatology of stress-triggered psychiatric and mood disorders (Robinson et al. 2012), one of which may be AUD.

The above-described differences at the behavioural and physiological levels were also associated with the differences in the expression of genes and proteins in several brain regions of interest. The main locus of differences between PF-insensitive and PF-sensitive rats was the Nacc, where differences in gene expression related to serotonergic (*Htr1a*, *Htr2a*, and *Slc6a4*), GABAergic (*Gabbr2*), glutamatergic (*Grm2*), and dopaminergic (*Slc6a3*) neurotransmission, as well as NPY neuromodulation, were revealed, and in some cases (5-HT2A and GABABR2) were also confirmed at the protein level. Considering the important role of serotonin in the mediation of impulsive actions observed in addiction, the differences in the components of the 5-HT system were not surprising. Indeed, preclinical research has shown that modulating activity at 5-HT2A receptors may block the expression of alcohol self-administration (Serra et al. 2022) and may also decrease the amount of alcohol intake (Berquist and Fantegrossi 2021). To our knowledge, however, this is the first study showing that differences in the expression of the 5-HT2A receptor can be associated with sensitivity to PF and, indirectly, with various aspects of alcohol addiction.

Similarly, the difference observed in the expression of the GABAB2 receptor, which in a number of previous studies was demonstrated to regulate alcohol sensitivity at the molecular and cellular levels, was not surprising (Farokhnia et al. 2018; Liang et al. 2006; Maccioni et al. 2010). Indeed, alterations in GABA signalling through pharmacological activation or deactivation of GABABRs were also shown to regulate behaviour and brain reward processes, as well as the reinforcing effects of drugs of abuse, including alcohol (Vlachou and Markou 2010). Analogous to 5-HT2A, the lower level of GABAB2 receptors in PF- insensitive animals suggests that decreased GABA signalling is linked to hyposensitivity to PF and stronger motivation to drink alcohol as well as proneness to reinstate drinking following a period of abstinence.

The second locus of the differences between the PF-insensitive and PF-sensitive rats was the Amy, where differences in the expression of genes related to dopaminergic (*Maob*) and GABAergic (*Gabbr1*) neurotransmission were revealed, and in the case of MAO-B, also confirmed at the protein level. Analyses of the intergroup differences in the level of MAO-B revealed a significant interaction between the effects of PF sensitivity and alcohol drinking. In animals sensitive to PF, the level of this enzyme was significantly higher in rats that consumed alcohol than in those that consumed water. Although MAO-B activity has been extensively investigated in alcoholism, there is a considerable inconsistency in the results. The finding of significantly higher MAO-B availability in PF-sensitive, alcohol-drinking animals is in line with some previous studies, which reported an increase in MAO-B levels and activity following chronic ethanol exposure (Ou et al. 2011; Zimatkin et al. 1997), but not with others, reporting no effects of alcohol consumption on MAO-B activity in rats (Della Corte et al. 1994; Sherif et al. 1993). Moreover, MAO-B has been believed to be involved in dopamine degradation, which supports the idea that the increased levels of this enzyme can be attributed to a decrease in extracellular dopamine concentration and enhanced sensitivity to rewarding feedback. However, the exact nature of the interaction between the level of MAO-B, PF sensitivity, and alcohol drinking should be unveiled by further studies.

Last but not least, significant, and confirmed at the protein level, differences in the expression of the *Adh1* gene were detected in the mPFC, and NAcc, where animals from the alcohol drinking group demonstrated significantly higher levels of this enzyme compared to their water drinking conspecifics. This result seems to validate the applied alcohol drinking procedure at the molecular level. Indeed, mammalian Adhs play a key role in alcohol metabolism and in the interindividual differences it exerts on the body (Edenberg 2007). Chronic alcohol abuse has been demonstrated to lead to Adh induction, increasing alcohol metabolism; thus, elevated levels of this enzyme in EtOH drinking groups confirm efficient exposure to chronic alcohol in our animal model.

### Conclusions and limitations

Based on the results of the present experiments, it seems that using rodent-based models, such as the preclinical PRL, can help to reveal neurobiological processes linked with reinforcement-based cognitive biases and their role in AUD. Although we hope this research has provided enough evidence to support the validity of the claim that sensitivity to PF can determine the trajectories of alcohol addiction, there are still a number of outstanding issues that future research will need to address. First, we still do not know the degree of the causal relationship between increased/decreased sensitivity to PF and vulnerability to AUD. Further development of translational preclinical tests of sensitivity to PF should help to elucidate this issue and may help to design personalized treatments based on these cognitive variables. Second, although we have demonstrated that there are distributed changes in physiological and molecular variables within multiple regions of the brain that occur over the course of alcohol use in rats and can persist into periods of abstinence, further studies looking at neurochemical correlates of altered feedback sensitivity in this context are needed. Although the WB method is one of the most reliable techniques for protein identification and quantification, its application is limited by the availability of high-quality specific primary antibodies against a given protein. For this reason, we were not able to confirm changes at the protein level in the expression of certain genes (Comt, Drd2, Gria1, Tph2, Grm2, and Npy), which could provide additional valuable insights into the changes induced by PF- sensitivity x alcohol interactions.

We also need more detailed pharmacological studies using drugs with known profiles in humans to understand the value of targeting PF sensitivity in AUD. It will be highly desirable to use voltammetry, optogenetics, or other biosensors and electrophysiological measures to characterize neuronal pathways and to elucidate the exact function and dynamic balance between cortical and subcortical regions involved in the interaction between PF sensitivity and AUD. Finally, further conceptual and empirical development is required to provide an integrated account of the role of PF sensitivity in the aetiology, development, and recurrence of AUD.

### Supplementary Information

Below is the link to the electronic supplementary material.

Supplementary file1 (DOCX 7871 KB)

## Data Availability

The data that support the findings of this study are available from the corresponding author, [RR], upon reasonable request.
