# Supplementary material for: Trait sensitivity to positive feedback is a predisposing factor for several aspects of compulsive alcohol drinking in male rats: behavioural, physiological, and molecular correlates
Source: Psychopharmacology (Berl). 2023 Sep 8;241(1):33–47. doi: 10.1007/s00213-023-06460-1 (PMC10774643; doi:10.1007/s00213-023-06460-1)
Supplement: Supplementary file 1 — Supplementary file1 (DOCX 7871 KB) [file 213_2023_6460_MOESM1_ESM.docx]

**Supplemental material**

**
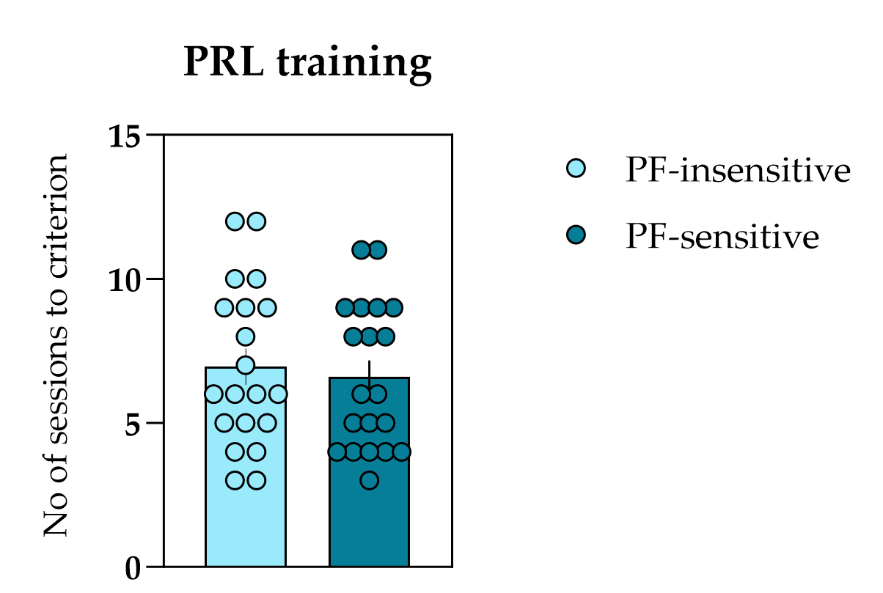
**

**Figure S1. PRL training.** The average number of tests needed to achieve PRL training criterion in PF-insensitive (N = 20) and PF-sensitive (N = 20) rats.

**
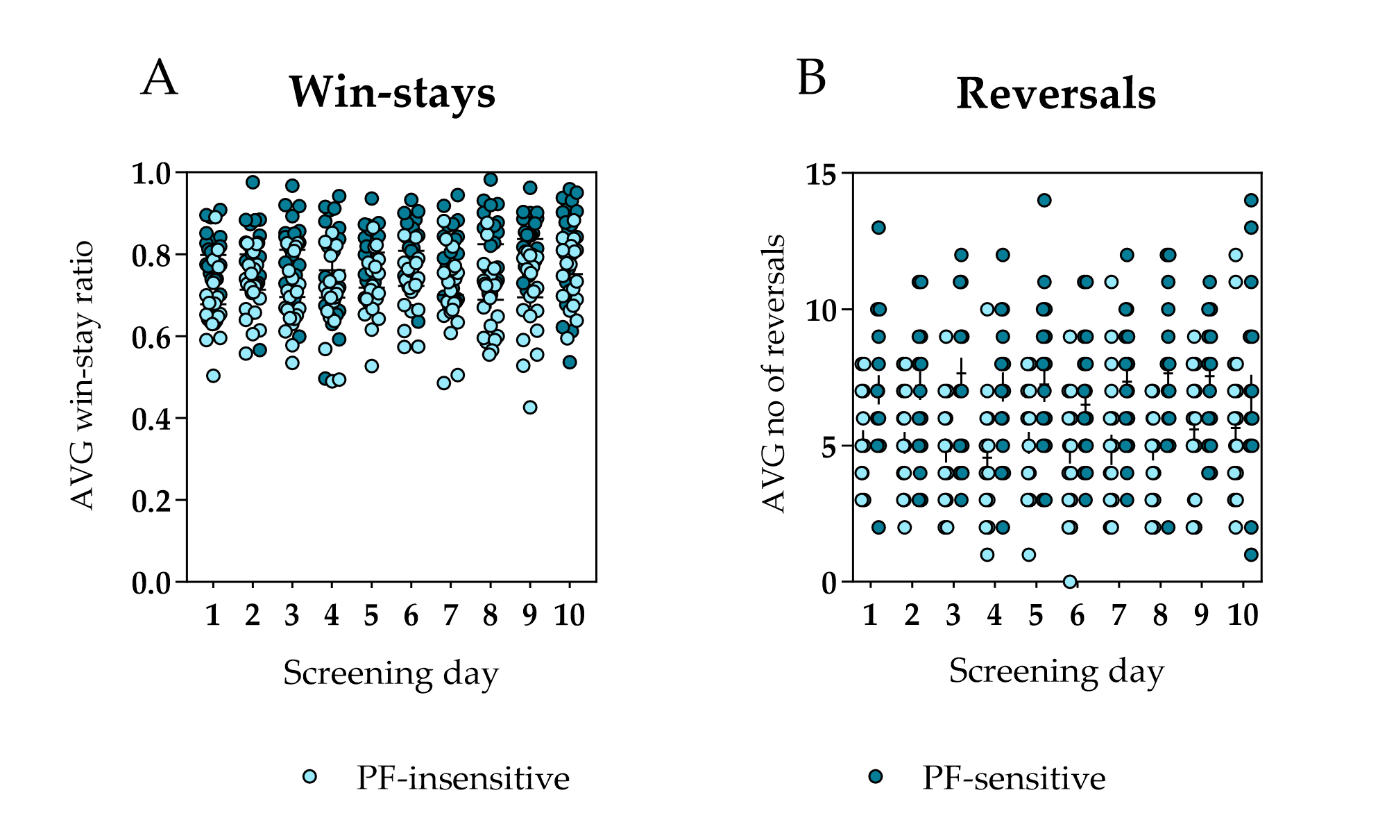
**

**Figure S2. Positive feedback sensitivity screening.** Individual data for (A) win-stay behaviours, and (B) reversal performance of all tested animals (20 PF-insensitive and 20 PF-sensitive) across 10 PRL screening tests.


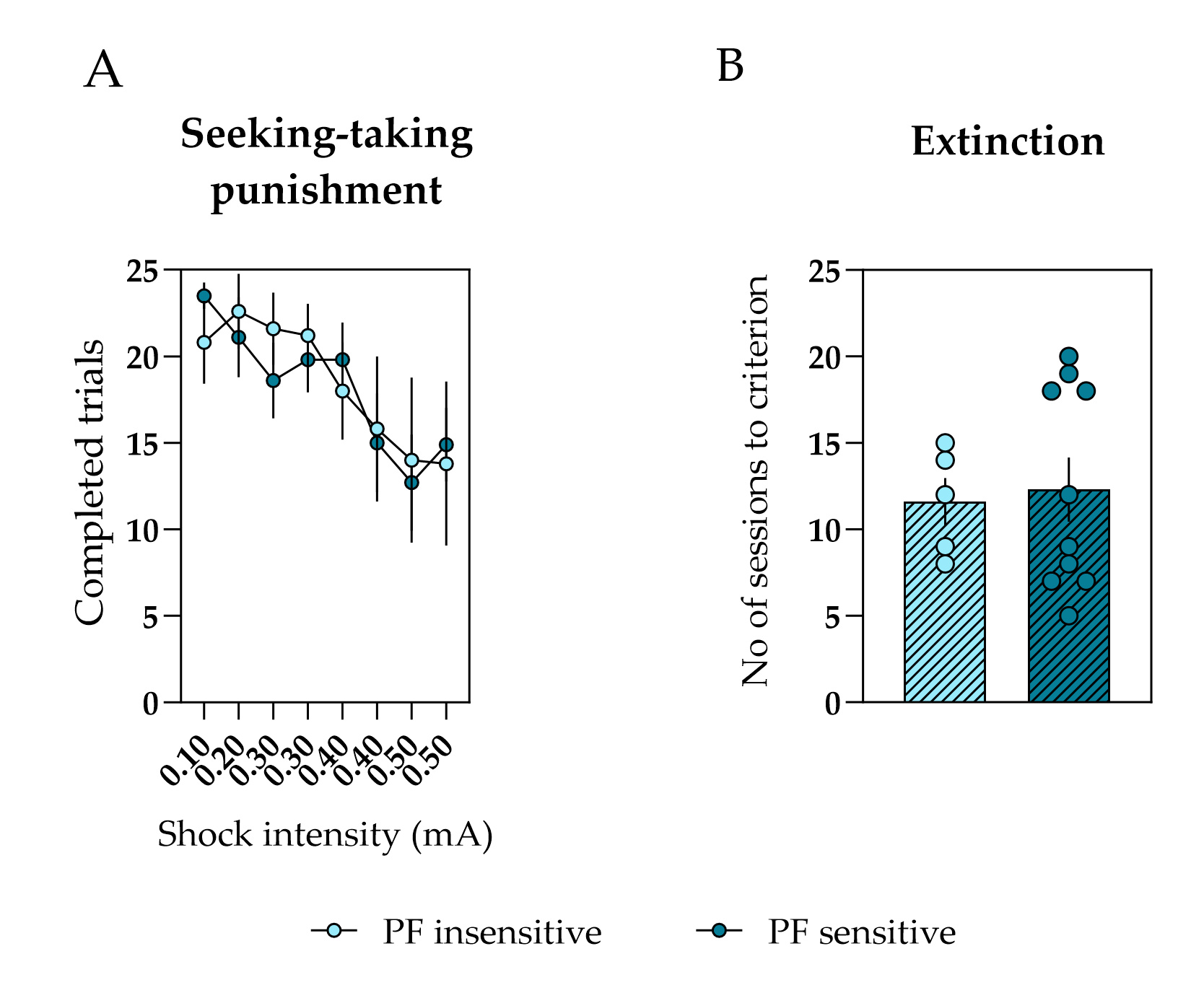


**Figure S3.** **The effects of trait sensitivity to positive feedback (PF) on the alcohol-seeking in face of electric shock punishment (A), and on the length of alcohol-seeking extinction (B) in rats.** A) As the shock intensity increased, the rats significantly decreased the number of completed trials. There were no significant differences in the number of trials completed between the PF-insensitive (N = 5) and PF-sensitive (N = 10) groups of animals. B) Following the seeking-taking punishment procedure, the rats underwent 5 baseline seeking-taking tests and were subsequently subjected to the extinction phase, during which seeking responses did not result in alcohol delivery. There was no statistical difference in length of extinction between PF-insensitive (N = 5) and PF-sensitive (N = 10) rats. Data are presented as the mean ± SEM

**Table S1. The effects of trait sensitivity to PF and alcohol drinking on gene expression.** Statistically significant effects and interactions are bolded.

| **Structure** | **Gene name** | **N** | **Interaction** | **Treatment** | **Sensitivity** |
| --- | --- | --- | --- | --- | --- |
| **ACC** | *Adh1* | 32 | F _(1, 28)_ = 0.988  p = 0.329 | F _(1, 28)_ = 3.627  p = 0.067 | F _(1, 28)_ = 0.019  p = 0.890 |
|  | *Cat* | 34 | F _(1, 30)_ = 0.019  p = 0.890 | F _(1, 30)_ = 1.185  p = 0.285 | F _(1, 30)_ = 0.035  p = 0.854 |
|  | ***Comt*** | 34 | F _(1, 30)_ = 0.387  p = 0.534 | **F _(1, 30)_ = 5.223**  p = **0.030 *** | F _(1, 30)_ = 0.020  p = 0.888 |
|  | ***Drd1*** | 33 | F _(1, 29)_ = 3.969  p = 0.056 | F _(1, 29)_ = 0.761  p = 0.390 | **F _(1, 29)_ = 4.556**  p = **0.041 *** |
|  | *Drd2* | 34 | F _(1, 30)_ = 0.282  p = 0.600 | F _(1, 30)_ = 1.521  p = 0.227 | F _(1, 30)_ = 0.349  p = 0.559 |
|  | *Gabbr1* | 34 | F _(1, 30)_ = 0.165  p = 0.688 | F _(1, 30)_ = 2.581  p = 0.119 | F _(1, 30)_ = 1.213  p = 0.280 |
|  | *Gabbr2* | 34 | F _(1, 30)_ = 0.001  p = 0.979 | F _(1, 30)_ = 0.673  p = 0.416 | F _(1, 30)_ = 0.052  p = 0.821 |
|  | *Gabra1* | 34 | F _(1, 30)_ = 0.899  p = 0.351 | F _(1, 30)_ = 0.512  p = 0.480 | F _(1, 30)_ = 0.611  p = 0.440 |
|  | *Gad1* | 34 | F _(1, 30)_ = 0.369  p = 0.548 | F _(1, 30)_ = 0.752  p = 0.393 | F _(1, 30)_ = 0.369  p = 0.548 |
|  | *Gad2* | 34 | F _(1, 30)_ = 0.467  p = 0.500 | F _(1, 30)_ = 0.230  p = 0.635 | F _(1, 30)_ = 0.343  p = 0.562 |
|  | ***Gria1*** | 34 | F _(1, 30)_ = 0.265  p = 0.611 | F _(1, 30)_ = 0.143  p = 0.708 | **F _(1, 30)_ = 4.809**  p = **0.036 *** |
|  | *Grin2a* | 34 | F _(1, 30)_ = 0.899  p = 0.351 | F _(1, 30)_ = 0.512  p = 0.480 | F _(1, 30)_ = 0.611  p = 0.441 |
|  | *Grin2b* | 34 | F _(1, 30)_ = 0.143  p = 0.708 | F _(1, 30)_ = 0.199  p = 0.660 | F _(1, 30)_ = 0.033  p = 0.857 |
|  | *Grm2* | 34 | F _(1, 30)_ = 0.396  p = 0.534 | F _(1, 30)_ = 0.031  p = 0.861 | F _(1, 30)_ = 3.754  p = 0.062 |
|  | *Grm3* | 34 | F _(1, 30)_ = 0.026  p = 0.873 | F _(1, 30)_ = 0.385  p = 0.539 | F _(1, 30)_ < 0.001  p = 0.985 |
|  | *Grm5* | 34 | F _(1, 30)_ = 0.074  p = 0.788 | F _(1, 30)_ = 1.217  p = 0.279 | F _(1, 30)_ = 2.077  p = 0.160 |
|  | *Htr1a* | 34 | F _(1, 30)_ = 0.053  p = 0.820 | F _(1, 30)_ = 2.300  p = 0.140 | F _(1, 30)_ = 1.634  p = 0.211 |
|  | *Htr2a* | 34 | F _(1, 30)_ = 1.209  p = 0.280 | F _(1, 30)_ = 0.012  p = 0.912 | F _(1, 30)_ = 1.607  p = 0.215 |
|  | *Htr2b* |  | - | | |
|  | ***Htr3a*** | 34 | F _(1, 30)_ = 0.104  p = 0.749 | F _(1, 30)_ = 0.126  p = 0.725 | **F _(1, 30)_ = 5.855**  **p = 0.022 *** |
|  | *Npy* | 34 | Kruskal‒Wallis test: p = 0.395 | | |
|  | ***Maoa*** | 33 | F _(1, 29)_ = 0.458  p = 0.504 | **F _(1, 29)_ = 4.732**  **p = 0.038 *** | F _(1, 29)_ = 0.231  p = 0.635 |
|  | *Maob* | 34 | F _(1, 30)_ = 1.643  p = 0.210 | F _(1, 30)_ = 1.510  p = 0.229 | F _(1, 30)_ = 0.371  p = 0.547 |
|  | *Slc1a2* | 34 | F _(1, 30)_ = 1.064  p = 0.311 | F _(1, 30)_ = 0.014  p = 0.907 | F _(1, 30)_ = 0.019  p = 0.892 |
|  | *Slc6a3* |  | - | | |
|  | *Slc6a4* | 32 | F _(1, 28)_ = 2.125  p = 0.156 | F _(1, 28)_ = 0.088  p = 0.769 | F _(1, 28)_ = 0.539  p = 0.469 |
|  | *Th* | 34 | F _(1, 30)_ = 0.235  p = 0.631 | F _(1, 30)_ = 1.553  p = 0.222 | F _(1, 30)_ = 0.773  p = 0.386 |
|  | *Tph2* | 34 | Kruskal‒Wallis test: p = 0.081 | | |
| **mPFC** | ***Adh1*** | **33** | **F _(1, 29)_ = 5.048**  **p = 0.032 *** | **F _(1, 29)_ = 5.072**  **p = 0.032 *** | F _(1, 29)_ = 3.423  p = 0.075 |
|  | ***Cat*** | 34 | F _(1, 30)_ = 0.037  p = 0.849 | **F _(1, 30)_ = 7.312**  **p = 0.011 *** | **F _(1, 30)_ = 9.431**  **p = 0.005 **** |
|  | ***Comt*** | 34 | F _(1, 30)_ = 0.447  p = 0.509 | **F _(1, 30)_ = 18.320**  **p < 0.001 ***** | F _(1, 30)_ = 2.791  p = 0.105 |
|  | *Drd1* | 34 | F _(1, 30)_ = 0.033  p = 0.856 | F _(1, 30)_ = 1.779  p = 0.193 | F _(1, 30)_ = 0.958  p = 0.336 |
|  | *Drd2* | 34 | F _(1, 29)_ = 0.194  p = 0.663 | F _(1, 29)_ = 0.517  p = 0.478 | F _(1, 29)_ = 0.336  p = 0.567 |
|  | *Gabbr1* | 34 | F _(1, 30)_ = 0.002  p = 0.965 | F _(1, 30)_ = 0.993  p = 0.327 | F _(1, 30)_ = 2.292  p = 0.141 |
|  | *Gabbr2* | 34 | Kruskal‒Wallis test: p = 0.609 | | |
|  | *Gabra1* | 34 | F _(1, 30)_ = 1.029  p = 0.318 | F _(1, 30)_ = 0.001  p = 0.975 | F _(1, 30)_ = 2.779  p = 0.106 |
|  | *Gad1* | 34 | F _(1, 30)_ = 0.266  p = 0.610 | F _(1, 30)_ = 1.918  p = 0.176 | F _(1, 30)_ = 2.092  p = 0.158 |
|  | *Gad2* | 34 | F _(1, 30)_ = 0.108  p = 0.745 | F _(1, 30)_ = 0.790  p = 0.379 | F _(1, 30)_ = 2.224  p = 0.146 |
|  | *Gria1* | 34 | F _(1, 30)_ = 0.001  p = 0.981 | F _(1, 30)_ = 0.702  p = 0.409 | F _(1, 30)_ = 0.154  p = 0.698 |
|  | *Grin2a* | 34 | Kruskal‒Wallis test: p = 0.092 | | |
|  | *Grin2b* | 34 | F _(1, 30)_ = 0.195  p = 0.662 | F _(1, 30)_ = 0.452  p = 0.506 | F _(1, 30)_ = 0.456  p = 0.505 |
|  | *Grm2* | 34 | F _(1, 30)_ = 0.056  p = 0.815 | F _(1, 30)_ = 0.386  p = 0.539 | F _(1, 30)_ = 4.144  p = 0.051 |
|  | *Grm3* | 34 | F _(1, 30)_ = 0.084  p = 0.774 | F _(1, 30)_ = 0.001  p = 0.974 | F _(1, 30)_ = 0.405  p = 0.530 |
|  | *Grm5* | 34 | Kruskal‒Wallis test: p = 0.989 | | |
|  | *Htr1a* | 34 | F _(1, 30)_ = 0.066  p = 0.799 | F _(1, 30)_ = 0.314  p = 0.580 | F _(1, 30)_ = 0.315  p = 0.579 |
|  | *Htr2a* | 34 | F _(1, 30)_ = 0.010  p = 0.921 | F _(1, 30)_ = 0.032  p = 0.859 | F _(1, 30)_ = 2.933  p = 0.097 |
|  | *Htr2b* | 29 | F _(1, 25)_ = 0.885  p = 0.356 | F _(1, 25)_ = 1.594  p = 0.218 | F _(1, 25)_ = 1.171  p = 0.290 |
|  | *Htr3a* | 34 | F _(1, 30)_ = 0.676  p = 0.4174 | F _(1, 30)_ = 0.716  p = 0.404 | F _(1, 30)_ = 0.828  p = 0.370 |
|  | *Npy* | 34 | F _(1, 30)_ = 0.806  p = 0.376 | F _(1, 30)_ = 3.297  p = 0.079 | F _(1, 30)_ = 1.166  p = 0.289 |
|  | *Maoa* | 34 | F _(1, 30)_ = 0.002  p = 0.968 | F _(1, 30)_ = 0.518  p = 0.477 | F _(1, 30)_ = 1.151  p = 0.292 |
|  | *Maob* | 34 | F _(1, 30)_ = 0.010  p = 0.919 | F _(1, 30)_ = 4.052  p = 0.053 | F _(1, 30)_ = 3.084  p = 0.089 |
|  | *Slc1a2* | 34 | F _(1, 30)_ = 0.005  p = 0.946 | F _(1, 30)_ = 1.438  p = 0.240 | F _(1, 30)_ = 3.653  p = 0.066 |
|  | *Slc6a3* | 19 | F _(1, 15)_ = 0.103  p = 0.753 | F _(1, 15)_ = 0.010  p = 0.921 | F _(1, 15)_ = 0.530  p = 0.478 |
|  | *Slc6a4* | 34 | F _(1, 30)_ = 0.001  p = 0.972 | F _(1, 30)_ = 0.012  p = 0.914 | F _(1, 30)_ = 1.105  p = 0.302 |
|  | *Th* | 34 | F _(1, 30)_ = 0.018  p = 0.895 | F _(1, 30)_ = 0.227  p = 0.637 | F _(1, 30)_ = 0.373  p = 0.546 |
|  | *Tph2* | 33 | F _(1, 29)_ = 0.633  p = 0.433 | F _(1, 29)_ = 1.428  p = 0.242 | F _(1, 29)_ = 1.136  p = 0.295 |
| **Amy** | *Adh1* | 32 | Kruskal‒Wallis test: p = 0.124 | | |
|  | *Cat* | 31 | F _(1, 27)_ = 0.019  p = 0.891 | F _(1, 27)_ = 0.842  p = 0.367 | F _(1, 27)_ = 0.650  p = 0.427 |
|  | *Comt* | 32 | Kruskal‒Wallis test: p = 0.274 | | |
|  | *Drd1* | 32 | F _(1, 28)_ = 0.680  p = 0.417 | F _(1, 28)_ = 0.008  p = 0.932 | F _(1, 28)_ = 1.007  p = 0.324 |
|  | ***Drd2*** | **32** | **F _(1, 28)_ = 6.200**  **p = 0.019 *** | **F _(1, 28)_ = 5.092**  **p = 0.032 *** | F _(1, 28)_ = 0.991  p = 0.328 |
|  | ***Gabbr1*** | **31** | **F _(1, 27)_ = 9.466**  p = **0.005 **** | F _(1, 27)_ = 3.727  p = 0.064 | F _(1, 27)_ = 1.761  p = 0.196 |
|  | *Gabbr2* | 32 | F _(1, 28)_ = 0.043  p = 0.837 | F _(1, 28)_ = 0.474  p = 0.497 | F _(1, 28)_ = 0.886  p = 0.355 |
|  | *Gabra1* | 32 | F _(1, 28)_ = 0.187  p = 0.669 | F _(1, 28)_ = 0.118  p = 0.734 | F _(1, 28)_ = 1.525  p = 0.227 |
|  | ***Gad1*** | 32 | F _(1, 28)_ = 0.017  p = 0.897 | **F _(1, 28)_ = 4.338**  **p = 0.047 *** | F _(1, 28)_ = 1.216  p = 0.280 |
|  | *Gad2* | 32 | F _(1, 28)_ = 0.902  p = 0.350 | F _(1, 28)_ = 3.643  p = 0.067 | F _(1, 28)_ = 2.088  p = 0.160 |
|  | *Gria1* | 32 | F _(1, 28)_ = 1.170  p = 0.287 | F _(1, 28)_ = 0.274  p = 0.605 | F _(1, 28)_ = 0.707  p = 0.408 |
|  | *Grin2a* | 32 | F _(1, 28)_ = 0.022  p = 0.884 | F _(1, 28)_ = 1.490  p = 0.232 | F _(1, 28)_ = 0.125  p = 0.727 |
|  | *Grin2b* | 32 | F _(1, 28)_ = 0.144  p = 0.707 | F _(1, 28)_ = 0.197  p = 0.661 | F _(1, 28)_ = 0.217  p = 0.645 |
|  | *Grm2* | 32 | F _(1, 28)_ = 1.206  p = 0.282 | F _(1, 28)_ = 0.423  p = 0.521 | F _(1, 28)_ = 2.257  p = 0.144 |
|  | *Grm3* | 31 | F _(1, 27)_ = 0.055  p = 0.816 | F _(1, 27)_ = 0.003  p = 0.955 | F _(1, 27)_ = 2.095  p = 0.159 |
|  | *Grm5* | 32 | F _(1, 28)_ = 0.664  p = 0.422 | F _(1, 28)_ = 2.104  p = 0.158 | F _(1, 28)_ = 0.201  p = 0.657 |
|  | *Htr1a* | 32 | F _(1, 28)_ = 0.109  p = 0.743 | F _(1, 28)_ = 2.959  p = 0.096 | F _(1, 28)_ = 0.073  p = 0.790 |
|  | *Htr2a* | 32 | F _(1, 28)_ = 1.209  p = 0.280 | F _(1, 28)_ = 0.012  p = 0.912 | F _(1, 28)_ = 1.607  p = 0.215 |
|  | *Htr2b* | 26 | F _(1, 22)_ = 0.363  p = 0.553 | F_(1, 22)_ = 2.826  p = 0.107 | F_(1, 22)_ = 2.376  p = 0.138 |
|  | *Htr3a* | 32 | F _(1, 28)_ = 0.274  p = 0.605 | F _(1, 28)_ = 3.046  p = 0.092 | F _(1, 28)_ = 2.541  p = 0.122 |
|  | *Npy* | 32 | F _(1, 28)_ = 0.393  p = 0.536 | F _(1, 28)_ = 0.622  p = 0.437 | F _(1, 28)_ = 1.464  p = 0.236 |
|  | *Maoa* | 32 | Kruskal‒Wallis test: p = 0.523 | | |
|  | ***Maob*** | 32 | F _(1, 28)_ = 0.347  p = 0.561 | F _(1, 28)_ = 0.583  p = 0.451 | **F _(1, 28)_ = 5.804**  **p = 0.023 *** |
|  | *Slc1a2* | 32 | F _(1, 28)_ = 0.238  p = 0.630 | F _(1, 28)_ = 1.004  p = 0.325 | F _(1, 28)_ = 2.490  p = 0.126 |
|  | *Slc6a3* |  |  | - |  |
|  | *Slc6a4* | 32 | F _(1, 28)_ = 0.424  p = 0.520 | F _(1, 28)_ = 0.720  p = 0.403 | F _(1, 28)_ = 3.774  p = 0.062 |
|  | *Th* | 32 | Kruskal‒Wallis test: p = 0.086 | | |
|  | ***Tph2*** | **32** | **F _(1, 28)_ = 4.732**  **p = 0.038 *** | F _(1, 28)_ = 2.625  p = 0.116 | F _(1, 28)_ = 0.989  p = 0.329 |
| **OFC** | ***Adh1*** | **34** | **Kruskal‒Wallis test: p = 0.038 *** | | |
|  | ***Cat*** | 34 | F _(1, 30)_ = 0.029  p = 0.867 | **F _(1, 30)_ = 5.351**  p = **0.028 *** | F _(1, 30)_ = 0.916  p = 0.346 |
|  | *Comt* | 34 | F _(1, 30)_ = 0.001  p = 0.972 | F _(1, 30)_ = 1.396  p = 0.247 | F _(1, 30)_ = 0.387  p = 0.538 |
|  | *Drd1* | 32 | F _(1, 28)_ = 0.077  p = 0.784 | F _(1, 28)_ = 1.024  p = 0.320 | F _(1, 28)_ = 0.236  p = 0.631 |
|  | *Drd2* | 33 | F _(1, 29)_ = 0.219  p = 0.643 | F _(1, 29)_ = 0.787  p = 0.382 | F _(1, 29)_ = 2.284  p = 0.142 |
|  | *Gabbr1* | 34 | F _(1, 30)_ = 0.230  p = 0.635 | F _(1, 30)_ = 0.050  p = 0.824 | F _(1, 30)_ = 0.846  p = 0.365 |
|  | *Gabbr2* | 34 | F _(1, 30)_ = 2.565  p = 0.120 | F _(1, 30)_ = 1.091  p = 0.305 | F _(1, 30)_ = 1.394  p = 0.247 |
|  | *Gabra1* | 34 | F _(1, 30)_ = 0.249  p = 0.621 | F _(1, 30)_ = 0.054  p = 0.818 | F _(1, 30)_ = 1.224  p = 0.277 |
|  | *Gad1* | 34 | F _(1, 30)_ = 1.199  p = 0.282 | F _(1, 30)_ = 3.242  p = 0.082 | F _(1, 30)_ = 2.275  p = 0.142 |
|  | ***Gad2*** | 34 | F _(1, 30)_ = 0.708  p = 0.407 | **F _(1, 30)_ = 6.329**  **p = 0.018 *** | F _(1, 30)_ = 2.448  p = 0.128 |
|  | *Gria1* | 34 | F _(1, 30)_ = 0.361  p = 0.553 | F _(1, 30)_ = 2.001 x 10^-7^  p = 1.00 | F _(1, 30)_ = 0.273  p = 0.606 |
|  | *Grin2a* | 34 | F _(1, 30)_ = 0.516  p = 0.478 | F _(1, 30)_ = 0.163  p = 0.690 | F _(1, 30)_ = 1.719  p = 0.200 |
|  | *Grin2b* | 34 | F _(1, 30)_ = 0.032  p = 0.860 | F _(1, 30)_ = 0.377  p = 0.544 | F _(1, 30)_ = 0.416  p = 0.524 |
|  | *Grm2* | 34 | F _(1, 30)_ = 0.707  p = 0.407 | F _(1, 30)_ = 0.116  p = 0.736 | F _(1, 30)_ = 2.909  p = 0.098 |
|  | *Grm3* | 34 | F _(1, 30)_ = 0.026  p = 0.873 | F _(1, 30)_ = 0.385  p = 0.540 | F _(1, 30)_ = 0.0003  p = 0.985 |
|  | *Grm5* | 34 | F _(1, 30)_ = 0.161  p = 0.691 | F _(1, 30)_ = 3.220  p = 0.083 | F _(1, 30)_ = 3.073  p = 0.090 |
|  | ***Htr1a*** | 34 | F _(1, 30)_ = 0.115  p = 0.737 | **F _(1, 30)_ = 6.362**  **p = 0.017 *** | F _(1, 30)_ = 2.551  p = 0.121 |
|  | *Htr2a* | 34 | F _(1, 30)_ = 0.694  p = 0.411 | F _(1, 30)_ = 0.424  p = 0.520 | F _(1, 30)_ = 3.298  p = 0.079 |
|  | *Htr2b* | 29 | F _(1, 25)_ = 0.542  p = 0.468 | F _(1, 25)_ = 2.634  p = 0.117 | F _(1, 25)_ = 0.265  p = 0.611 |
|  | *Htr3a* | 34 | F _(1, 30)_ = 0.004  p = 0.953 | F _(1, 30)_ = 2.734  p = 0.109 | F _(1, 30)_ = 0.763  p = 0.390 |
|  | *Npy* | 33 | F _(1, 29)_ = 0.244  p = 0.625 | F _(1, 29)_ = 0.563  p = 0.459 | F _(1, 29)_ = 0.739  p = 0.397 |
|  | *Maoa* | 34 | F _(1, 30)_ = 1.889  p = 0.180 | F _(1, 30)_ = 1.091  p = 0.305 | F _(1, 30)_ = 3.852  p = 0.060 |
|  | *Maob* | 34 | F _(1, 30)_ = 0.070  p = 0.794 | F _(1, 30)_ = 0.320  p = 0.576 | F _(1, 30)_ = 0.099  p = 0.755 |
|  | *Slc1a2* | 34 | F _(1, 30)_ = 0.080  p = 0.780 | F _(1, 30)_ = 0.858  p = 0.362 | F _(1, 30)_ = 1.532  p = 0.225 |
|  | *Slc6a3* | 33 | F _(1, 29)_ = 0.909  p = 0.384 | F _(1, 29)_ = 0.123  p = 0.728 | F _(1, 29)_ = 0.136  p = 0.715 |
|  | *Slc6a4* | 33 | F _(1, 29)_ = 0.223  p = 0.641 | F _(1, 29)_ = 1.130  p = 0.297 | F _(1, 29)_ = 0.036  p = 0.851 |
|  | *Th* | 34 | F _(1, 30)_ = 2.040  p = 0.164 | F _(1, 30)_ = 0.146  p = 0.705 | F _(1, 30)_ = 1.342  p = 0.256 |
|  | *Tph2* | 34 | F _(1, 30)_ = 0.170  p = 0.683 | F _(1, 30)_ = 0.001  p = 0.981 | F _(1, 30)_ = 0.652  p = 0.426 |
| **Nacc** | ***Adh1*** | 31 | F _(1, 27)_ = 1.814  p = 0.189 | **F _(1, 27)_ = 8.590**  **p = 0.007 **** | F _(1, 27)_ = 4.137  p = 0.052 |
|  | *Cat* | 34 | F _(1, 30)_ = 0.409  p = 0.527 | F _(1, 30)_ = 0.005  p = 0.946 | F _(1, 30)_ = 0.819  p = 0.373 |
|  | *Comt* | 34 | F _(1, 30)_ = 0.314  p = 0.580 | F _(1, 30)_ = 0.839  p = 0.367 | F _(1, 30)_ = 0.458  p = 0.504 |
|  | *Drd1* | 34 | F _(1, 30)_ = 0.271  p = 0.607 | F _(1, 30)_ = 0.030  p = 0.864 | F _(1, 30)_ = 0.151  p = 0.701 |
|  | *Drd2* | 34 | F _(1, 30)_ = 0.046  p = 0.832 | F _(1, 30)_ = 0.1174  p = 0.734 | F _(1, 30)_ = 0.538  p = 0.469 |
|  | *Gabbr1* | 34 | F _(1, 30)_ = 0.126  p = 0.725 | F _(1, 30)_ = 0.003  p = 0.960 | F _(1, 30)_ = 0.966  p = 0.333 |
|  | ***Gabbr2*** | 33 | F _(1, 29)_ = 2.290  p = 0.141 | F _(1, 29)_ = 0.589  p = 0.449 | **F _(1, 29)_ = 6.557**  **p = 0.016 *** |
|  | *Gabra1* | 34 | F _(1, 30)_ = 3.502  p = 0.071 | F _(1, 30)_ = 0.268  p = 0.609 | F _(1, 30)_ = 1.199  p = 0.282 |
|  | *Gad1* | 34 | F _(1, 30)_ = 0.079  p = 0.781 | F _(1, 30)_ = 0.402  p = 0.531 | F _(1, 30)_ = 0.013  p = 0.909 |
|  | *Gad2* | 34 | F _(1, 30)_ = 0.460  p = 0.503 | F _(1, 30)_ = 1.212  p = 0.280 | F _(1, 30)_ = 0.786  p = 0.382 |
|  | *Gria1* | 34 | F _(1, 30)_ = 0.057  p = 0.812 | F _(1, 30)_ = 0.048  p = 0.829 | F _(1, 30)_ = 0.143  p = 0.708 |
|  | *Grin2a* | 34 | F _(1, 30)_ = 1.472  p = 0.235 | F _(1, 30)_ = 0.045  p = 0.833 | F _(1, 30)_ = 1.308  p = 0.262 |
|  | *Grin2b* | 34 | F _(1, 30)_ = 0.434  p = 0.515 | F _(1, 30)_ = 0.467  p = 0.500 | F _(1, 30)_ = 1.145  p = 0.293 |
|  | ***Grm2*** | 33 | F _(1, 29)_ = 1.724  p = 0.200 | F _(1, 29)_ = 0.184  p = 0.671 | **F _(1, 29)_ = 4.863**  **p = 0.036 *** |
|  | *Grm3* | 34 | F _(1, 30)_ = 0.219  p = 0.643 | F _(1, 30)_ = 0.125  p = 0.726 | F _(1, 30)_ = 0.458  p = 0.504 |
|  | *Grm5* | 34 | F _(1, 30)_ = 1.366  p = 0.252 | F _(1, 30)_ = 0.263  p = 0.612 | F _(1, 30)_ = 0.271  p = 0.607 |
|  | ***Htr1a*** | 34 | F _(1, 30)_ = 0.585  p = 0.450 | F _(1, 30)_ = 0.031  p = 0.863 | **F _(1, 30)_ = 6.452**  p = **0.017 *** |
|  | ***Htr2a*** | **34** | **F _(1, 30)_ = 4.606**  **p = 0.040 *** | F _(1, 30)_ = 2.248  p = 0.144 | **F _(1, 30)_ = 4.367**  **p = 0.045 *** |
|  | *Htr2b* | 23 | F _(1, 19)_ = 1.425  p = 0.247 | F _(1, 19)_ = 0.643  p = 0.433 | F _(1, 19)_ = 0.063  p = 0.804 |
|  | *Htr3a* | 34 | F _(1, 30)_ = 0.012  p = 0.913 | F _(1, 30)_ = 0.082  p = 0.776 | F _(1, 30)_ = 0.574  p = 0.455 |
|  | ***Npy*** | 34 | F _(1, 30)_ = 0.793  p = 0.380 | F _(1, 30)_ = 0.063  p = 0.804 | **F _(1, 30)_ = 10.020**  **p = 0.004 **** |
|  | *Maoa* | 34 | Kruskal‒Wallis test: p = 0.972 | | |
|  | *Maob* | 34 | F _(1, 30)_ = 0.182  p = 0.673 | F _(1, 30)_ = 0.502  p = 0.484 | F _(1, 30)_ = 0.0004  p = 0.983 |
|  | *Slc1a2* | 34 | F _(1, 30)_ = 0.009  p = 0.926 | F _(1, 30)_ = 0.002  p = 0.965 | F _(1, 30)_ = 0.061  p = 0.807 |
|  | ***Slc6a3*** | 34 | F _(1, 30)_ = 0.973  p = 0.332 | F _(1, 30)_ = 2.639  p = 0.115 | **F _(1, 30)_ = 5.166**  **p = 0.030 *** |
|  | ***Slc6a4*** | **33** | **F _(1, 29)_ = 4.977**  p = **0.034 *** | **F _(1, 29)_ = 6.895**  **p = 0.014 *** | F _(1, 29)_ = 0.134  p = 0.717 |
|  | *Th* | 33 | F _(1, 29)_ = 0.027  p = 0.871 | F _(1, 29)_ = 0.411  p = 0.527 | F _(1, 29)_ = 1.548  p = 0.223 |
|  | *Tph2* | 34 | F _(1, 30)_ = 0.174  p = 0.680 | F _(1, 30)_ = 0.020  p = 0.890 | F _(1, 30)_ = 0.013  p = 0.908 |

Abnormalities in the gene expression readings were detected in certain samples on the RT-PCR card, and these results were not included in the analysis.

| **Structure** | **Protein** | **N** | **Interaction** | **Treatment** | **Sensitivity** |
| --- | --- | --- | --- | --- | --- |
| **ACC** | DRD1 | 27 | F_(1, 23)_ = 0.384  p = 0.542 | F_(1, 23)_ = 2.454  p = 0.131 | F_(1, 23)_ = 1.364  p = 0.255 |
|  | 5-HT3A | 34 | F_(1, 30)_ = 0.009  p = 0.925 | F_(1, 30)_ = 1.495  p = 0.231 | F_(1, 30)_ = 0.08  p = 0.778 |
| **mPFC** | **ADH1** | 33 | F_(1, 29)_ = 1.333  p = 0.258 | **F_(1, 29)_ = 9.059**  **p = 0.005 **** | F_(1, 29)_ = 0.569  p = 0.457 |
|  | CAT | 33 | F_(1, 29)_ = 0.051  p = 0.822 | F_(1, 29)_ = 0.005  p = 0.945 | F_(1, 29)_ = 0.113  p = 0. 739 |
| **Amy** | **MAO-B** | **34** | **F_(1, 30)_ = 7.650**  **p = 0.010 **** | F_(1, 30)_ = 0.104  p = 0.750 | F_(1, 30)_ = 0.135  p = 0. 716 |
| **OFC** | **ADH1** | 34 | F_(1, 30)_ = 0.063  p = 0.803 | **F_(1, 30)_ = 4.753**  **p = 0.037 *** | F_(1, 30)_ = 0.906  p = 0.349 |
| **Nacc** | **ADH1** | 34 | F_(1, 30)_ = 3.572  p = 0.069 | **F_(1, 30)_ = 7.287**  **p = 0.011 *** | F_(1, 30)_ = 0.015  p = 0.904 |
|  | **GABABR2** | 32 | F_(1, 28)_ = 0.084  p = 0.774 | F_(1, 28)_ = 3.136  p = 0.088 | **F_(1, 28)_ = 5.422**  **p = 0.027 *** |
|  | 5-HT1A | 33 | F_(1, 29)_ = 0.017  p = 0.896 | F_(1, 29)_ = 2.306  p = 0.140 | F_(1, 29)_ = 0.358  p = 0. 554 |
|  | **5-HT2A** | 34 | F_(1, 30)_ = 3.495  p = 0.071 | F_(1, 30)_ = 2.384  p = 0.133 | **F_(1, 30)_ = 6.689**  **p = 0.015 *** |

***Table S2. Western blot analysis.*** *Statistically significant effects and interactions are bolded*

Some protein bands were unsuitable for quantification due to a technical error.

**Original Western blot images used for quantification of protein levels**

Legend:

I: PF-insensitive, S: PF-sensitive, bands that have not been quantified due to technical reasons are marked with an arrow.

**ADH1_mPFC**

ADH1_mPFC_membrane 1

| **Treatment** | **EtOH** | | | | | | **H2O** | | | | | | | |
| --- | --- | --- | --- | --- | --- | --- | --- | --- | --- | --- | --- | --- | --- | --- |
| **PF sensitivity** | **I** | **I** | **S** | **S** | **I** | **S** | **S** | **S** | **S** | **S** | **S** | **I** | **I** | **I** |
| **Rat’s nr** | **3** | **7** | **1** | **2** | **17** | **4** | **12** | **18** | **13** | **15** | **20** | **10** | **25** | **26** |





**40 kDa**

βactin_mPFC_membrane 1 (ADH1)

| **Treatment** | **EtOH** | | | | | | **H2O** | | | | | | | |
| --- | --- | --- | --- | --- | --- | --- | --- | --- | --- | --- | --- | --- | --- | --- |
| **PF sensitivity** | **I** | **I** | **S** | **S** | **I** | **S** | **S** | **S** | **S** | **S** | **S** | **I** | **I** | **I** |
| **Rat’s nr** | **3** | **7** | **1** | **2** | **17** | **4** | **12** | **18** | **13** | **15** | **20** | **10** | **25** | **26** |





**42 kDa**

ADH1_mPFC_membrane 2

| **Treatment** | **EtOH** | | | | | | | **H2O** | | | | | | |
| --- | --- | --- | --- | --- | --- | --- | --- | --- | --- | --- | --- | --- | --- | --- |
| **PF sensitivity** | **S** | **S** | **S** | **S** | **I** | **I** | **I** | **I** | **I** | **I** | **S** | **S** | **S** | **S** |
| **Rat’s nr** | **2** | **5** | **9** | **29** | **17** | **19** | **16** | **22** | **28** | **36** | **30** | **24** | **38** | **39** |





**40 kDa**

βactin_mPFC_membrane 2 (ADH1)

| **Treatment** | **EtOH** | | | | | | | **H2O** | | | | | | |
| --- | --- | --- | --- | --- | --- | --- | --- | --- | --- | --- | --- | --- | --- | --- |
| **PF sensitivity** | **S** | **S** | **S** | **S** | **I** | **I** | **I** | **I** | **I** | **I** | **S** | **S** | **S** | **S** |
| **Rat’s nr** | **2** | **5** | **9** | **29** | **17** | **19** | **16** | **22** | **28** | **36** | **30** | **24** | **38** | **39** |





**42 kDa**

ADH1_mPFC_membrane 3

| **Treatment** | **EtOH** | | | | | | | **H2O** | | | | | | |
| --- | --- | --- | --- | --- | --- | --- | --- | --- | --- | --- | --- | --- | --- | --- |
| **PF sensitivity** | **S** | **S** | **S** | **S** | **I** | **I** | **I** | **S** | **S** | **S** | **I** | **I** | **S** | **I** |
| **Rat’s nr** | **33** | **35** | **21** | **12** | **19** | **7** | **3** | **6** | **11** | **30** | **27** | **31** | **14** | **10** |





**40 kDa**

βactin_mPFC_membrane 3 (ADH1)

| **Treatment** | **EtOH** | | | | | | | **H2O** | | | | | | |
| --- | --- | --- | --- | --- | --- | --- | --- | --- | --- | --- | --- | --- | --- | --- |
| **PF sensitivity** | **S** | **S** | **S** | **S** | **I** | **I** | **I** | **S** | **S** | **S** | **I** | **I** | **S** | **I** |
| **Rat’s nr** | **33** | **35** | **21** | **12** | **19** | **7** | **3** | **6** | **11** | **30** | **27** | **31** | **14** | **10** |





**42 kDa**

**MAOB_AMY**

MAOB_AMY_membrane 1

| **Treatment** | **EtOH** | | | | | | **H2O** | | | | | | | |
| --- | --- | --- | --- | --- | --- | --- | --- | --- | --- | --- | --- | --- | --- | --- |
| **PF sensitivity** | **I** | **I** | **S** | **S** | **I** | **S** | **S** | **S** | **S** | **S** | **S** | **I** | **I** | **I** |
| **Rat’s nr** | **3** | **7** | **1** | **2** | **17** | **4** | **12** | **18** | **13** | **15** | **20** | **10** | **25** | **26** |





**60 kDa**

βactin_AMY_membrane 1 (MAOB)

| **Treatment** | **EtOH** | | | | | | **H2O** | | | | | | | |
| --- | --- | --- | --- | --- | --- | --- | --- | --- | --- | --- | --- | --- | --- | --- |
| **PF sensitivity** | **I** | **I** | **S** | **S** | **I** | **S** | **S** | **S** | **S** | **S** | **S** | **I** | **I** | **I** |
| **Rat’s nr** | **3** | **7** | **1** | **2** | **17** | **4** | **12** | **18** | **13** | **15** | **20** | **10** | **25** | **26** |





**42 kDa**

MAOB_AMY_membrane 2

| **Treatment** | **EtOH** | | | | | | | **H2O** | | | | | | |
| --- | --- | --- | --- | --- | --- | --- | --- | --- | --- | --- | --- | --- | --- | --- |
| **PF sensitivity** | **S** | **S** | **S** | **S** | **I** | **I** | **I** | **I** | **I** | **I** | **S** | **S** | **S** | **S** |
| **Rat’s nr** | **2** | **5** | **9** | **29** | **17** | **19** | **16** | **22** | **28** | **36** | **30** | **24** | **38** | **39** |





**60 kDa**

βactin_AMY_membrane 2 (MAOB)

| **Treatment** | **EtOH** | | | | | | | **H2O** | | | | | | |
| --- | --- | --- | --- | --- | --- | --- | --- | --- | --- | --- | --- | --- | --- | --- |
| **PF sensitivity** | **S** | **S** | **S** | **S** | **I** | **I** | **I** | **I** | **I** | **I** | **S** | **S** | **S** | **S** |
| **Rat’s nr** | **2** | **5** | **9** | **29** | **17** | **19** | **16** | **22** | **28** | **36** | **30** | **24** | **38** | **39** |





**42 kDa**

MAOB_AMY_membrane 3

| **Treatment** | **EtOH** | | | | | | | **H2O** | | | | | | |
| --- | --- | --- | --- | --- | --- | --- | --- | --- | --- | --- | --- | --- | --- | --- |
| **PF sensitivity** | **S** | **S** | **S** | **S** | **I** | **I** | **I** | **S** | **S** | **S** | **I** | **I** | **S** | **I** |
| **Rat’s nr** | **33** | **35** | **21** | **12** | **19** | **7** | **3** | **6** | **11** | **30** | **27** | **31** | **14** | **10** |





**60 kDa**

βactin_AMY_membrane 3 (MAOB)

| **Treatment** | **EtOH** | | | | | | | **H2O** | | | | | | |
| --- | --- | --- | --- | --- | --- | --- | --- | --- | --- | --- | --- | --- | --- | --- |
| **PF sensitivity** | **S** | **S** | **S** | **S** | **I** | **I** | **I** | **S** | **S** | **S** | **I** | **I** | **S** | **I** |
| **Rat’s nr** | **33** | **35** | **21** | **12** | **19** | **7** | **3** | **6** | **11** | **30** | **27** | **31** | **14** | **10** |





**42 kDa**

**ADH1_OFC**

ADH1_OFC_membrane 1

| **Treatment** | **EtOH** | | | | | | **H2O** | | | | | | | |
| --- | --- | --- | --- | --- | --- | --- | --- | --- | --- | --- | --- | --- | --- | --- |
| **PF sensitivity** | **I** | **I** | **S** | **S** | **I** | **S** | **S** | **S** | **S** | **S** | **S** | **I** | **I** | **I** |
| **Rat’s nr** | **3** | **7** | **1** | **2** | **17** | **4** | **12** | **18** | **13** | **15** | **20** | **10** | **25** | **26** |





**40 kDa**

βactin_OFC_membrane 1 (ADH1)

| **Treatment** | **EtOH** | | | | | | **H2O** | | | | | | | |
| --- | --- | --- | --- | --- | --- | --- | --- | --- | --- | --- | --- | --- | --- | --- |
| **PF sensitivity** | **I** | **I** | **S** | **S** | **I** | **S** | **S** | **S** | **S** | **S** | **S** | **I** | **I** | **I** |
| **Rat’s nr** | **3** | **7** | **1** | **2** | **17** | **4** | **12** | **18** | **13** | **15** | **20** | **10** | **25** | **26** |





**42 kDa**

ADH1_OFC_membrane 2

| **Treatment** | **EtOH** | | | | | | | **H2O** | | | | | | |
| --- | --- | --- | --- | --- | --- | --- | --- | --- | --- | --- | --- | --- | --- | --- |
| **PF sensitivity** | **S** | **S** | **S** | **S** | **I** | **I** | **I** | **I** | **I** | **I** | **S** | **S** | **S** | **S** |
| **Rat’s nr** | **2** | **5** | **9** | **29** | **17** | **19** | **16** | **22** | **28** | **36** | **30** | **24** | **38** | **39** |





**40 kDa**

βactin_OFC_membrane 2 (ADH1)

| **Treatment** | **EtOH** | | | | | | | **H2O** | | | | | | |
| --- | --- | --- | --- | --- | --- | --- | --- | --- | --- | --- | --- | --- | --- | --- |
| **PF sensitivity** | **S** | **S** | **S** | **S** | **I** | **I** | **I** | **I** | **I** | **I** | **S** | **S** | **S** | **S** |
| **Rat’s nr** | **2** | **5** | **9** | **29** | **17** | **19** | **16** | **22** | **28** | **36** | **30** | **24** | **38** | **39** |





**42 kDa**

ADH1_OFC_membrane 3

| **Treatment** | **EtOH** | | | | | | | **H2O** | | | | | | |
| --- | --- | --- | --- | --- | --- | --- | --- | --- | --- | --- | --- | --- | --- | --- |
| **PF sensitivity** | **S** | **S** | **S** | **S** | **I** | **I** | **I** | **S** | **S** | **S** | **I** | **I** | **S** | **I** |
| **Rat’s nr** | **33** | **35** | **21** | **12** | **19** | **7** | **3** | **6** | **11** | **30** | **27** | **31** | **14** | **10** |





**40 kDa**

βactin_OFC_membrane 3 (ADH1)

| **Treatment** | **EtOH** | | | | | | | **H2O** | | | | | | |
| --- | --- | --- | --- | --- | --- | --- | --- | --- | --- | --- | --- | --- | --- | --- |
| **PF sensitivity** | **S** | **S** | **S** | **S** | **I** | **I** | **I** | **S** | **S** | **S** | **I** | **I** | **S** | **I** |
| **Rat’s nr** | **33** | **35** | **21** | **12** | **19** | **7** | **3** | **6** | **11** | **30** | **27** | **31** | **14** | **10** |





**42 kDa**

**ADH1_Nacc**

ADH1_Nacc_membrane 1

| **Treatment** | **EtOH** | | | | | | **H2O** | | | | | | | |
| --- | --- | --- | --- | --- | --- | --- | --- | --- | --- | --- | --- | --- | --- | --- |
| **PF sensitivity** | **I** | **I** | **S** | **S** | **I** | **S** | **S** | **S** | **S** | **S** | **S** | **I** | **I** | **I** |
| **Rat’s nr** | **3** | **7** | **1** | **2** | **17** | **4** | **12** | **18** | **13** | **15** | **20** | **10** | **25** | **26** |





**40 kDa**

βactin_Nacc_membrane 1 (ADH1)

| **Treatment** | **EtOH** | | | | | | **H2O** | | | | | | | |
| --- | --- | --- | --- | --- | --- | --- | --- | --- | --- | --- | --- | --- | --- | --- |
| **PF sensitivity** | **I** | **I** | **S** | **S** | **I** | **S** | **S** | **S** | **S** | **S** | **S** | **I** | **I** | **I** |
| **Rat’s nr** | **3** | **7** | **1** | **2** | **17** | **4** | **12** | **18** | **13** | **15** | **20** | **10** | **25** | **26** |





**42 kDa**

ADH1_Nacc_membrane 2

| **Treatment** | **EtOH** | | | | | | | **H2O** | | | | | | |
| --- | --- | --- | --- | --- | --- | --- | --- | --- | --- | --- | --- | --- | --- | --- |
| **PF sensitivity** | **S** | **S** | **S** | **S** | **I** | **I** | **I** | **I** | **I** | **I** | **S** | **S** | **S** | **S** |
| **Rat’s nr** | **2** | **5** | **9** | **29** | **17** | **19** | **16** | **22** | **28** | **36** | **30** | **24** | **38** | **39** |





**40 kDa**

βactin_Nacc_membrane 2 (ADH1)

| **Treatment** | **EtOH** | | | | | | | **H2O** | | | | | | |
| --- | --- | --- | --- | --- | --- | --- | --- | --- | --- | --- | --- | --- | --- | --- |
| **PF sensitivity** | **S** | **S** | **S** | **S** | **I** | **I** | **I** | **I** | **I** | **I** | **S** | **S** | **S** | **S** |
| **Rat’s nr** | **2** | **5** | **9** | **29** | **17** | **19** | **16** | **22** | **28** | **36** | **30** | **24** | **38** | **39** |





**42 kDa**

ADH1_Nacc_membrane 3

| **Treatment** | **EtOH** | | | | | | | **H2O** | | | | | | |
| --- | --- | --- | --- | --- | --- | --- | --- | --- | --- | --- | --- | --- | --- | --- |
| **PF sensitivity** | **S** | **S** | **S** | **S** | **I** | **I** | **I** | **S** | **S** | **S** | **I** | **I** | **S** | **I** |
| **Rat’s nr** | **33** | **35** | **21** | **12** | **19** | **7** | **3** | **6** | **11** | **30** | **27** | **31** | **14** | **10** |





**40 kDa**

βactin_Nacc_membrane 3 (ADH1)

| **Treatment** | **EtOH** | | | | | | | **H2O** | | | | | | |
| --- | --- | --- | --- | --- | --- | --- | --- | --- | --- | --- | --- | --- | --- | --- |
| **PF sensitivity** | **S** | **S** | **S** | **S** | **I** | **I** | **I** | **S** | **S** | **S** | **I** | **I** | **S** | **I** |
| **Rat’s nr** | **33** | **35** | **21** | **12** | **19** | **7** | **3** | **6** | **11** | **30** | **27** | **31** | **14** | **10** |





**42 kDa**

**GABABR2_Nacc**

GABABR2_Nacc_membrane 1

| **Treatment** | **EtOH** | | | | | | **H2O** | | | | | | | |
| --- | --- | --- | --- | --- | --- | --- | --- | --- | --- | --- | --- | --- | --- | --- |
| **PF sensitivity** | **I** | **I** | **S** | **S** | **I** | **S** | **S** | **S** | **S** | **S** | **S** | **I** | **I** | **I** |
| **Rat’s nr** | **3** | **7** | **1** | **2** | **17** | **4** | **12** | **18** | **13** | **15** | **20** | **10** | **25** | **26** |





**90 kDa**

βactin_Nacc_membrane 1 (GABABR2)

| **Treatment** | **EtOH** | | | | | | **H2O** | | | | | | | |
| --- | --- | --- | --- | --- | --- | --- | --- | --- | --- | --- | --- | --- | --- | --- |
| **PF sensitivity** | **I** | **I** | **S** | **S** | **I** | **S** | **S** | **S** | **S** | **S** | **S** | **I** | **I** | **I** |
| **Rat’s nr** | **3** | **7** | **1** | **2** | **17** | **4** | **12** | **18** | **13** | **15** | **20** | **10** | **25** | **26** |





**42 kDa**

GABABR2_Nacc_membrane 2

| **Treatment** | **EtOH** | | | | | | | **H2O** | | | | | | |
| --- | --- | --- | --- | --- | --- | --- | --- | --- | --- | --- | --- | --- | --- | --- |
| **PF sensitivity** | **S** | **S** | **S** | **S** | **I** | **I** | **I** | **I** | **I** | **I** | **S** | **S** | **S** | **S** |
| **Rat’s nr** | **2** | **5** | **9** | **29** | **17** | **19** | **16** | **22** | **28** | **36** | **30** | **24** | **38** | **39** |





**90 kDa**

βactin_Nacc_membrane 2 (GABABR2)

| **Treatment** | **EtOH** | | | | | | | **H2O** | | | | | | |
| --- | --- | --- | --- | --- | --- | --- | --- | --- | --- | --- | --- | --- | --- | --- |
| **PF sensitivity** | **S** | **S** | **S** | **S** | **I** | **I** | **I** | **I** | **I** | **I** | **S** | **S** | **S** | **S** |
| **Rat’s nr** | **2** | **5** | **9** | **29** | **17** | **19** | **16** | **22** | **28** | **36** | **30** | **24** | **38** | **39** |





**42 kDa**

GABABR2_Nacc_membrane 3

| **Treatment** | **EtOH** | | | | | | | **H2O** | | | | | | |
| --- | --- | --- | --- | --- | --- | --- | --- | --- | --- | --- | --- | --- | --- | --- |
| **PF sensitivity** | **S** | **S** | **S** | **S** | **I** | **I** | **I** | **S** | **S** | **S** | **I** | **I** | **S** | **I** |
| **Rat’s nr** | **33** | **35** | **21** | **12** | **19** | **7** | **3** | **6** | **11** | **30** | **27** | **31** | **14** | **10** |





**90 kDa**

βactin_Nacc_membrane 3 (GABABR2)

| **Treatment** | **EtOH** | | | | | | | **H2O** | | | | | | |
| --- | --- | --- | --- | --- | --- | --- | --- | --- | --- | --- | --- | --- | --- | --- |
| **PF sensitivity** | **S** | **S** | **S** | **S** | **I** | **I** | **I** | **S** | **S** | **S** | **I** | **I** | **S** | **I** |
| **Rat’s nr** | **33** | **35** | **21** | **12** | **19** | **7** | **3** | **6** | **11** | **30** | **27** | **31** | **14** | **10** |





**42 kDa**

**SERT_Nacc**

SERT_Nacc_membrane 1

| **Treatment** | **EtOH** | | | | | | **H2O** | | | | | | | |
| --- | --- | --- | --- | --- | --- | --- | --- | --- | --- | --- | --- | --- | --- | --- |
| **PF sensitivity** | **I** | **I** | **S** | **S** | **I** | **S** | **S** | **S** | **S** | **S** | **S** | **I** | **I** | **I** |
| **Rat’s nr** | **3** | **7** | **1** | **2** | **17** | **4** | **12** | **18** | **13** | **15** | **20** | **10** | **25** | **26** |


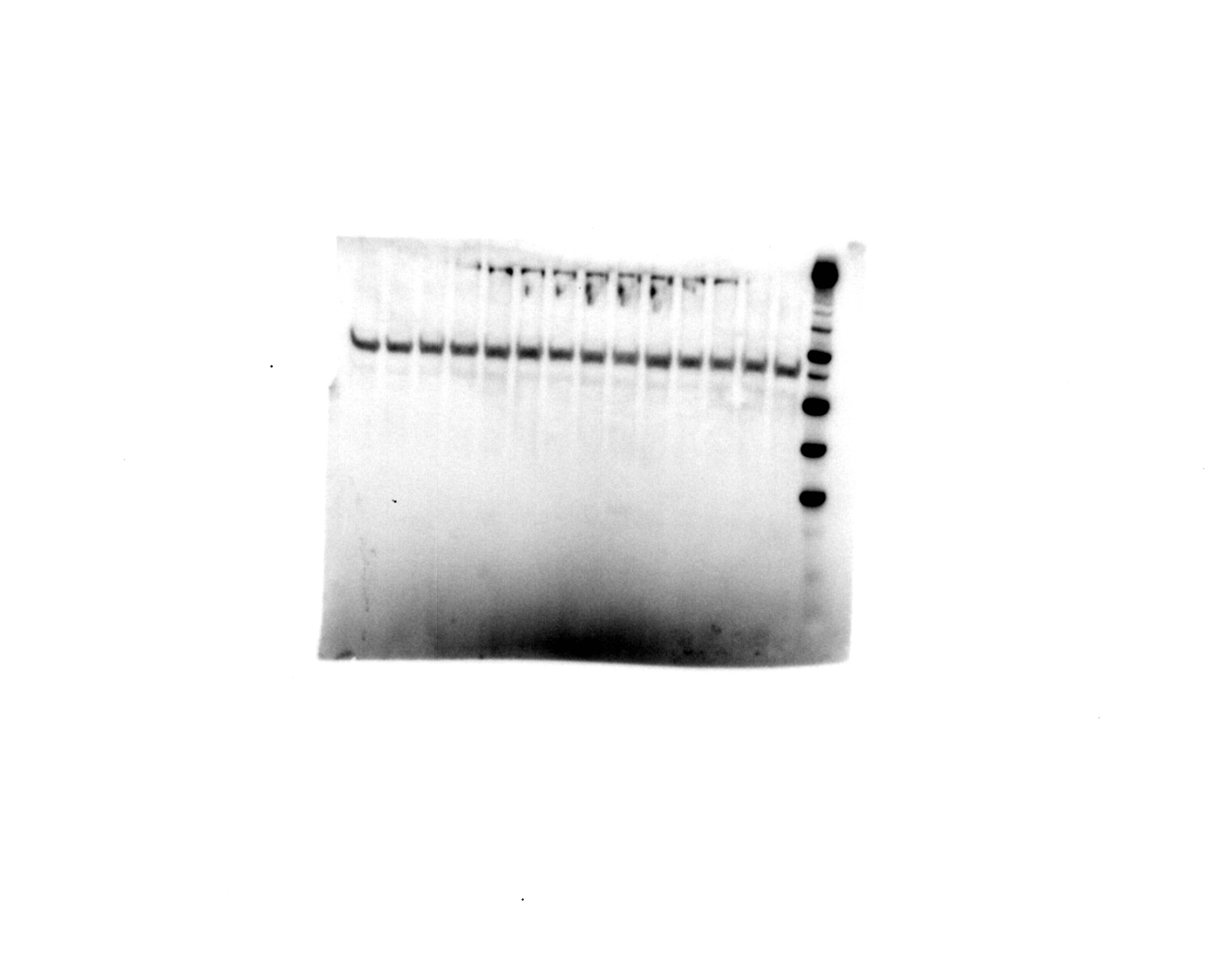


**80 kDa**

βactin_Nacc_membrane 1 (SERT)

| **Treatment** | **EtOH** | | | | | | **H2O** | | | | | | | |
| --- | --- | --- | --- | --- | --- | --- | --- | --- | --- | --- | --- | --- | --- | --- |
| **PF sensitivity** | **I** | **I** | **S** | **S** | **I** | **S** | **S** | **S** | **S** | **S** | **S** | **I** | **I** | **I** |
| **Rat’s nr** | **3** | **7** | **1** | **2** | **17** | **4** | **12** | **18** | **13** | **15** | **20** | **10** | **25** | **26** |


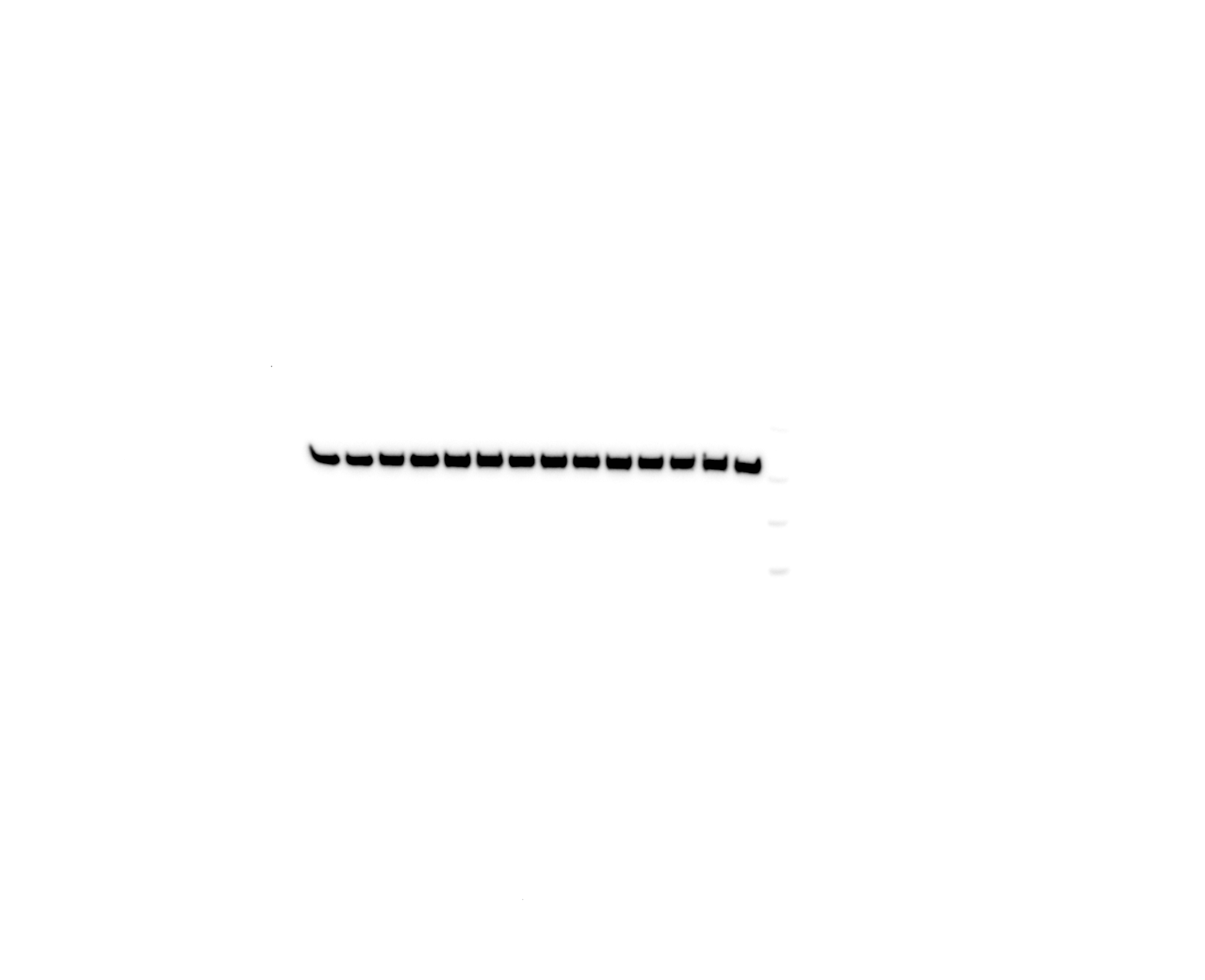


**42 kDa**

SERT_Nacc_membrane 2

| **Treatment** | **EtOH** | | | | | | | **H2O** | | | | | | |
| --- | --- | --- | --- | --- | --- | --- | --- | --- | --- | --- | --- | --- | --- | --- |
| **PF sensitivity** | **S** | **S** | **S** | **S** | **I** | **I** | **I** | **I** | **I** | **I** | **S** | **S** | **S** | **S** |
| **Rat’s nr** | **2** | **5** | **9** | **29** | **17** | **19** | **16** | **22** | **28** | **36** | **30** | **24** | **38** | **39** |


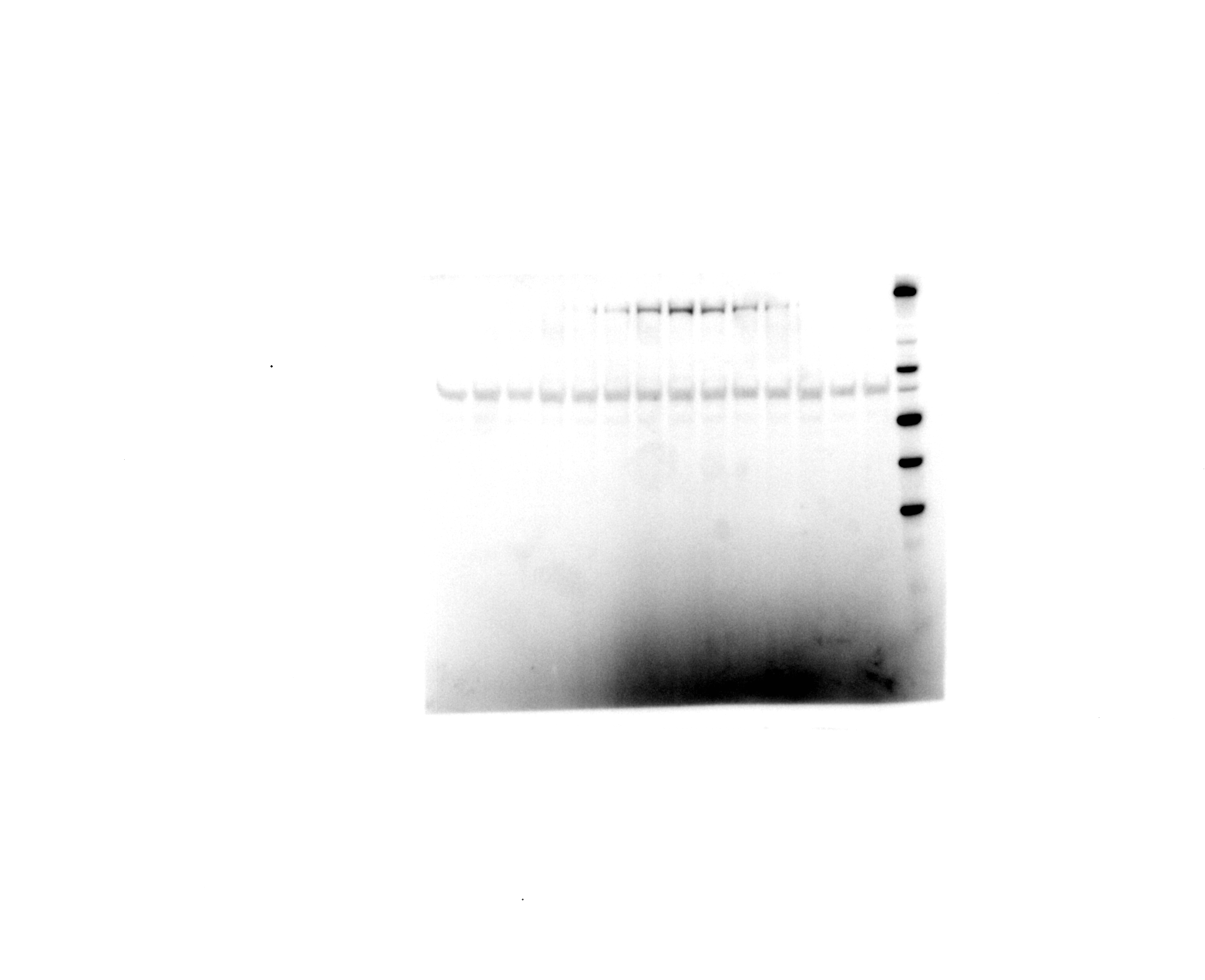


**80 kDa**

βactin_Nacc_membrane 1 (SERT)

| **Treatment** | **EtOH** | | | | | | | **H2O** | | | | | | |
| --- | --- | --- | --- | --- | --- | --- | --- | --- | --- | --- | --- | --- | --- | --- |
| **PF sensitivity** | **S** | **S** | **S** | **S** | **I** | **I** | **I** | **I** | **I** | **I** | **S** | **S** | **S** | **S** |
| **Rat’s nr** | **2** | **5** | **9** | **29** | **17** | **19** | **16** | **22** | **28** | **36** | **30** | **24** | **38** | **39** |


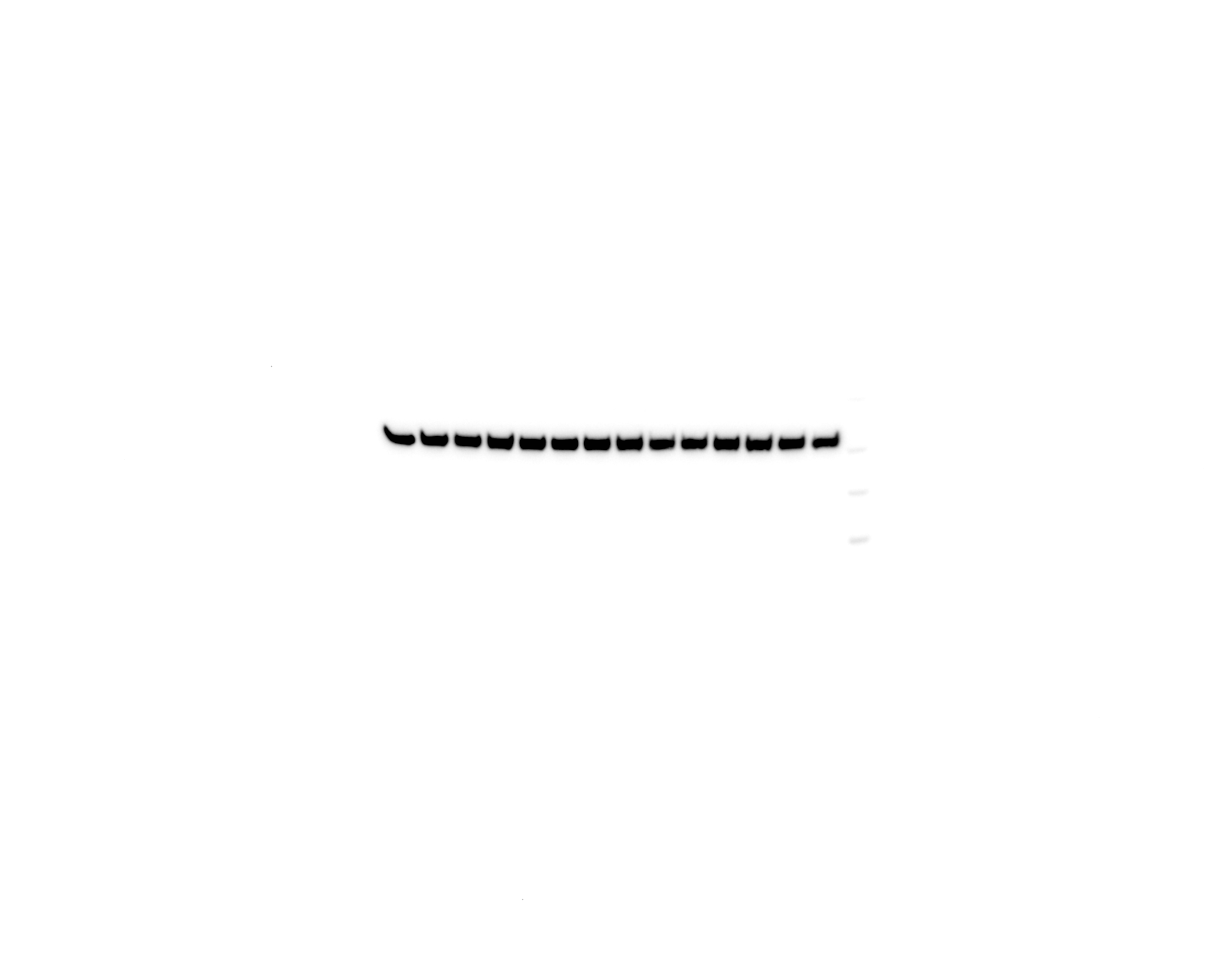


**42 kDa**

SERT_Nacc_membrane 3

| **Treatment** | **EtOH** | | | | | | | **H2O** | | | | | | |
| --- | --- | --- | --- | --- | --- | --- | --- | --- | --- | --- | --- | --- | --- | --- |
| **PF sensitivity** | **S** | **S** | **S** | **S** | **I** | **I** | **I** | **S** | **S** | **S** | **I** | **I** | **S** | **I** |
| **Rat’s nr** | **33** | **35** | **21** | **12** | **19** | **7** | **3** | **6** | **11** | **30** | **27** | **31** | **14** | **10** |





**80 kDa**

βactin_Nacc_membrane 3 (SERT)

| **Treatment** | **EtOH** | | | | | | | **H2O** | | | | | | |
| --- | --- | --- | --- | --- | --- | --- | --- | --- | --- | --- | --- | --- | --- | --- |
| **PF sensitivity** | **S** | **S** | **S** | **S** | **I** | **I** | **I** | **S** | **S** | **S** | **I** | **I** | **S** | **I** |
| **Rat’s nr** | **33** | **35** | **21** | **12** | **19** | **7** | **3** | **6** | **11** | **30** | **27** | **31** | **14** | **10** |


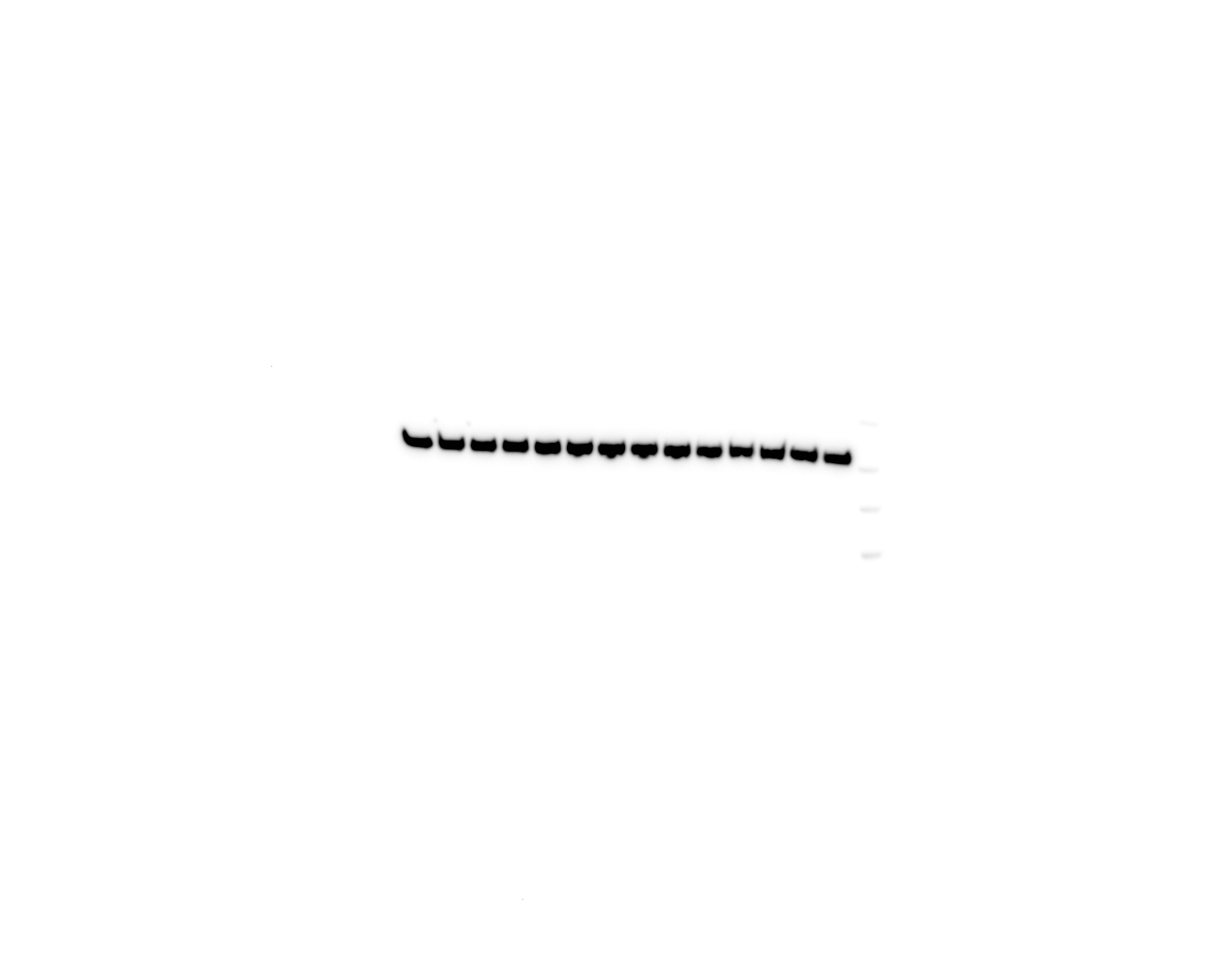


**42 kDa**

**5-HT2A_Nacc**

5-HT2A_Nacc_membrane 1

| **Treatment** | **EtOH** | | | | | | **H2O** | | | | | | | |
| --- | --- | --- | --- | --- | --- | --- | --- | --- | --- | --- | --- | --- | --- | --- |
| **PF sensitivity** | **I** | **I** | **S** | **S** | **I** | **S** | **S** | **S** | **S** | **S** | **S** | **I** | **I** | **I** |
| **Rat’s nr** | **3** | **7** | **1** | **2** | **17** | **4** | **12** | **18** | **13** | **15** | **20** | **10** | **25** | **26** |





**53 kDa**

βactin_Nacc_membrane 1 (5-HT2A)

| **Treatment** | **EtOH** | | | | | | **H2O** | | | | | | | |
| --- | --- | --- | --- | --- | --- | --- | --- | --- | --- | --- | --- | --- | --- | --- |
| **PF sensitivity** | **I** | **I** | **S** | **S** | **I** | **S** | **S** | **S** | **S** | **S** | **S** | **I** | **I** | **I** |
| **Rat’s nr** | **3** | **7** | **1** | **2** | **17** | **4** | **12** | **18** | **13** | **15** | **20** | **10** | **25** | **26** |





**42 kDa**

5-HT2A_Nacc_membrane 2

| **Treatment** | **EtOH** | | | | | | | **H2O** | | | | | | |
| --- | --- | --- | --- | --- | --- | --- | --- | --- | --- | --- | --- | --- | --- | --- |
| **PF sensitivity** | **S** | **S** | **S** | **S** | **I** | **I** | **I** | **I** | **I** | **I** | **S** | **S** | **S** | **S** |
| **Rat’s nr** | **2** | **5** | **9** | **29** | **17** | **19** | **16** | **22** | **28** | **36** | **30** | **24** | **38** | **39** |





**53 kDa**

βactin_Nacc_membrane 2 (5-HT2A)

| **Treatment** | **EtOH** | | | | | | | **H2O** | | | | | | |
| --- | --- | --- | --- | --- | --- | --- | --- | --- | --- | --- | --- | --- | --- | --- |
| **PF sensitivity** | **S** | **S** | **S** | **S** | **I** | **I** | **I** | **I** | **I** | **I** | **S** | **S** | **S** | **S** |
| **Rat’s nr** | **2** | **5** | **9** | **29** | **17** | **19** | **16** | **22** | **28** | **36** | **30** | **24** | **38** | **39** |





**42 kDa**

5-HT2A_Nacc_membrane 3

| **Treatment** | **EtOH** | | | | | | | **H2O** | | | | | | |
| --- | --- | --- | --- | --- | --- | --- | --- | --- | --- | --- | --- | --- | --- | --- |
| **PF sensitivity** | **S** | **S** | **S** | **S** | **I** | **I** | **I** | **S** | **S** | **S** | **I** | **I** | **S** | **I** |
| **Rat’s nr** | **33** | **35** | **21** | **12** | **19** | **7** | **3** | **6** | **11** | **30** | **27** | **31** | **14** | **10** |





**53 kDa**

βactin_Nacc_membrane 3 (5-HT2A)

| **Treatment** | **EtOH** | | | | | | | **H2O** | | | | | | |
| --- | --- | --- | --- | --- | --- | --- | --- | --- | --- | --- | --- | --- | --- | --- |
| **PF sensitivity** | **S** | **S** | **S** | **S** | **I** | **I** | **I** | **S** | **S** | **S** | **I** | **I** | **S** | **I** |
| **Rat’s nr** | **33** | **35** | **21** | **12** | **19** | **7** | **3** | **6** | **11** | **30** | **27** | **31** | **14** | **10** |





**42 kDa**
